# Full genome sequencing, evolutionary dynamics, and pathogenicity evaluation of chicken infectious anemia virus with emphasis on Upper Egypt reveals genetic variability linked to vaccinal strains

**DOI:** 10.1186/s12985-026-03257-4

**Published:** 2026-07-24

**Authors:** Eman Abd Elmenum Shosha, Ibrahim Eldaghayes, Ali Mahmoud Zanaty, Mohammed A. Gamaleldin, Mervat Masoud Mohamed, A. N. Gamal Maha, Doha Abd Alrahman Ahmed

**Affiliations:** 1 Virology Department, Faculty of Veterinary Medicine, New Valley University, El-Khargia, 72511 Egypt; 2 Department of Microbiology and Parasitology, Faculty of Veterinary Medicine, University of Tripoli, P.O. Box 13662, Tripoli, Libya; 3 Reference Laboratory for Quality Control On Poultry, Agriculture Research Center (ARC), Animal Health Institute (AHRI), Giza, 12619 Egypt; 4 Department of Poultry Disease, Animal Health Research Institute, Agricultural Research Center, Assiut, 71526 Egypt; 5 Central Laboratory for Evaluation of Veterinary Biologics (CLEVB), Agricultural Research Center (ARC), Cairo, 11381 Egypt; 6 Department of Avian and Rabbit Medicine, Faculty of Veterinary Medicine, Assiut University, Assiut, Egypt

**Keywords:** CIAV, Genotype II, Recombination, 3D Modeling, Upper Egypt, Pathogenicity

## Abstract

Despite intensive vaccination efforts, *Chicken Infectious Anemia Virus* (CIAV) remains a formidable threat to the Egyptian poultry industry, primarily through the emergence of vaccine-escape variants. While most studies have focused on Northern Egypt, there is a critical knowledge gap regarding the viral landscape in the Southern provinces. This study provides the most extensive surveillance to date, spanning 10 diverse Egyptian governorates with a strategic focus on Upper Egypt (2024–2025). Out of 400 collected samples, TaqMan-based qPCR revealed a high prevalence of 30% (120/400). Five representative isolates were selected for whole-genome sequencing and molecular characterization. The isolates were classified as Genotype II by phylogenetic analysis, showing a notable genetic divergence (85–87% amino acid similarity) from traditional Genotype I vaccine strains. Notably, comprehensive recombination analysis identified isolate PX96998 as a mosaic variant, with breakpoints indicating intra-genotypic recombination between closely related genotype II lineages, highlighting complex evolutionary dynamics in the field. Furthermore, 3D homology modeling of the VP1 protein identified critical structural alterations in functional loop regions, potentially hindering the binding affinity of neutralizing antibodies and facilitating immune evasion. In an experimental setting, the genotype II isolate demonstrated hypervirulence in SPF chickens, causing severe development retardation and extensive atrophy of lymphoid organs (p < 0.0001). Pathognomonic pale, fatty bone marrow with a sharp drop in PCV% to 18 ± 0.58% indicated severe aplastic anemia. By the fifth week after infection, there was a complete humoral collapse with undetectable ELISA titers due to the huge systemic replication revealed by viral load measurement, which peaked in the thymus (9.50 ± 0.25 log₁₀) and bone marrow (9.00 ± 0.22 log₁₀). These results were supported by histopathology, which revealed intranuclear inclusion bodies and systemic lymphocyte depletion. These findings support the highly pathogenic genotype II CIAV’s dominance and aggressive evolution in Egypt. The emergence of recombinant variants with altered structural epitopes underscores the urgent need to replace outdated vaccination plans with genotype-matched immunogens in order to lessen the severe consequences on Egypt’s poultry industry.

**Supplementary Information:**

The online version contains supplementary material available at https://doi.org/10.1186/s12985-026-03257-4.

## Introduction

The poultry industry in Egypt faces a continuous challenge from immunosuppressive pathogens, with chicken infectious anemia virus (CIAV) remaining at the forefront of these threats. As a major poultry pathogen, CIAV induces a state of severe immunopathology by targeting the primary lymphoid and hematopoietic tissues, leading to a cascade of economic losses due to secondary infections and compromised vaccine efficacy [1, 2]. According to the current International Committee on Taxonomy of Viruses (ICTV) classification system, CIAV is taxonomically classified under the realm *Floreoviria*, kingdom *Shotokuvirae*, phylum *Commensaviricota*, class *Cardeaviricetes*, order *Sanitavirales*, family *Anelloviridae*, genus *Gyrovirus*, and species *Gyrovirus chicken anemia* [3]. The virus possesses a single-stranded, closed circular DNA genome of approximately 2.3 kb [3]. The three mains partially overlapping open reading frames (ORFs) in the CAV genome are called VP1, VP2, and VP3. Their nucleotide sequence lengths are 1350, 651, and 366, respectively. VP1 (51.6 KDa), VP2 (211 KDa), and VP3 (13.6 KDa) are three viral proteins with varying molecular weights that are encoded by these three overlapping ORFs. The primary structural protein of the virus is called VP1, the scaffolding protein is called VP2, and the non-structural protein called apoptin is called VP3 [4, 5]. Neutralizing antibodies are induced by the protective proteins VP1 and VP2 [6]. Since the VP1 exhibits the greatest nucleotide diversity, it is typically employed for molecular research and genetic characterization of CAV [3–6].

Phylogenetic study based on the VP1 gene has identified three genetically distinct genotypes of CAV (I, II, and III). CAV isolates from various regions of the world have shown only slight differences in expressed amino acid sequences [3]. Previous investigations had only found a single serotype [7] until a possible serotype 2 strain (strain CAV-7) from the United States emerged [8]. Affected birds may exhibit clinical or subclinical symptoms, with young chicks under four weeks being the most susceptible. Key manifestations include increased mortality, slowed growth, and severe anemia, alongside pathognomonic lesions such as thymus and bone marrow atrophy, subcutaneous hemorrhages, and hemorrhagic syndrome commonly known as Blue Wing Disease [3, 6, 9]. At the cellular level, the virus targets lymphoid cells, inducing apoptosis triggered by VP3 expression. This subsequent lymphocyte depletion leads to significant immunosuppression, making the birds more vulnerable to secondary bacterial and viral infections [3]. The virus primarily targets hematopoietic cells in the bone marrow and T lymphoblasts within the thymus. This pathogenesis results in significant clinical impact among susceptible young chicks, with morbidity and mortality rates potentially climbing to 55% and 80%, respectively [2–9].

CAV exhibits a specific tropism, replicating in embryonated eggs and lymphoid cell lines but not in primary chicken cells like kidney cells or fibroblasts [10]. While MDCC-MSB1 suspension cultures are commonly employed for viral growth in molecular research [9], the labor-intensive nature of traditional isolation has shifted the focus toward rapid diagnostics. Consequently, PCR and hybridization are now routinely used to identify viral DNA directly from infected tissues [3–6]. Epidemiologically, being non-enveloped, CIAV is remarkably resistant to common disinfectants and environmental stressors, necessitating the use of potent, specialized disinfection protocols [11]. Furthermore, the epidemiology of the virus is complicated by its dual transmission routes: horizontal and vertical. Vertical transmission via the eggs of infected breeders is especially critical, as it leads to early-age infections and peak mortality in young chicks. Consequently, vaccinating breeder flocks is of paramount importance to provide progeny with protective maternal antibodies [6].

The chronological detection of CAV in Egypt began in 1990 in a commercial chicken farm. Historically, Egyptian poultry populations were primarily affected by Genotype I strains. Field isolates shared high genetic similarity with the CUX-1 vaccine strain, despite the presence of specific mutations associated with pathogenicity [3, 12]. However, the epidemiological landscape has recently shifted; [6, 13] have highlighted the emergence and rising dominance of Genotype II variants. This evolutionary transition suggests that the traditional Genotype I-based vaccines, such as Cux-1, may offer inadequate cross-protection. The genetic drift observed in the VP1 capsid protein’s hypervariable regions likely facilitates immune evasion, resulting in the clinical breakthroughs currently seen in Egyptian flocks.

Crucially, while several studies have characterized CIAV in Northern Egypt, there is a profound lack of molecular and pathogenic data concerning the strains circulating in Upper Egypt. This regional data scarcity has critically masked an ongoing, unmonitored viral evolution driven by distinct field conditions, environmental stressors, and unique localized vaccination pressures. It is highly hypothesized that these regional factors have accelerated the selection of distinct genetic variants capable of escaping standard vaccine-induced immunity, thereby directly explaining the recurrent vaccination failures observed in the field. The present study was therefore conducted to bridge this knowledge gap, providing the first comprehensive investigation into the prevalence, genetic evolution, and pathogenicity of CIAV in this geographically distinct and high-density poultry sector.

## Materials and methods

The present study was conducted in strict compliance with the established guidelines for the care and use of laboratory animals. All experimental procedures received prior approval from the Institutional Animal Care and Use Committee (IACUC) of the Faculty of Veterinary Medicine, Assiut University **(**Approval No. 02–3-10–2025). To alleviate pain and distress, birds were humanely anesthetized with a ketamine/xylazine mixture (25/5 mg/kg BW) before invasive procedures. Upon study completion, birds were humanely euthanized via cervical dislocation under deep general anesthesia, adhering to international animal welfare protocols.

## Epidemiological investigation and clinical characterization of suspected CIAV cases in egyptian poultry flocks

**Fig. 1:**
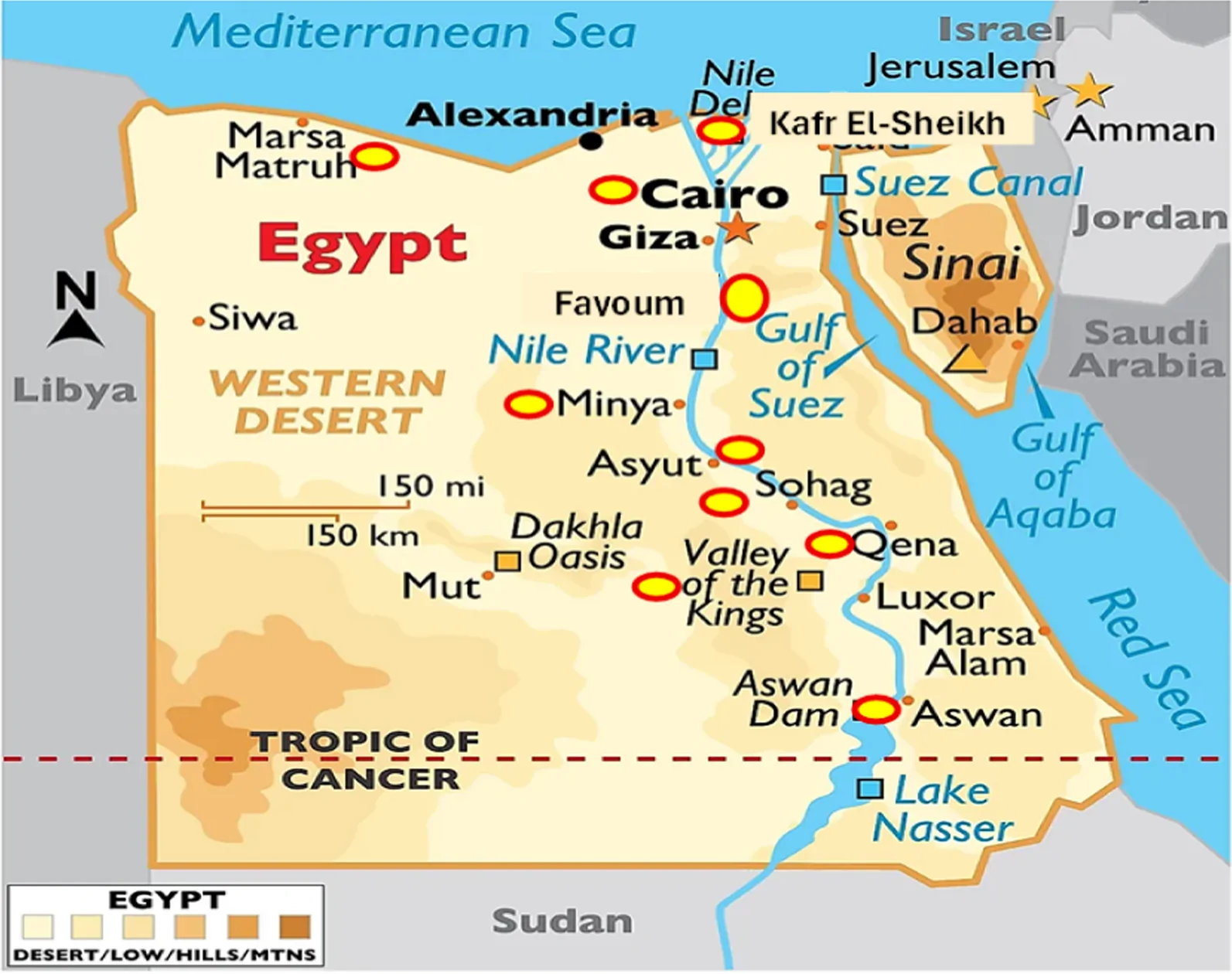
Geographical distribution of sampled Egyptian governorates (highlighted with yellow circles) showing positive cases of Chicken Infectious Anemia Virus during the period from April 2024 to November 2025

A comprehensive epidemiological survey was conducted over a 20-month period, from April 2024 to November 2025. A total of 400 clinical tissue samples, including the bursa of Fabricius, thymus, spleen, and liver, were collected from 80 different poultry flocks. The sampling strategy encompassed both commercial poultry farms and backyard (household) rearing systems across 10 Egyptian governorates, providing wide geographical coverage from Upper to Lower Egypt, including Assiut, Sohag, Aswan, New Valley, Qena, Minya, Fayoum, Cairo, Kafr El-Sheikh, and Matrouh (Table S1, Supplementary Material; Fig. 1). The study evaluated various poultry production sectors, including breeders, commercial layers, and broilers, with bird ages ranging between 2 and 27 weeks. Vaccination protocols differed across sectors: breeders and commercial layers received a single dose of the live-attenuated Cux-1 strain via drinking water (administered at 13–15 weeks), while broilers and backyard flocks remained unvaccinated. Clinically, the investigated birds showed signs indicative of CIAV, such as generalized weakness, pale combs, and stunted growth, with field mortality fluctuating between 4 and 15%. Significant gross lesions observed during necropsy included pale, watery bone marrow, marked atrophy of the thymus and bursa of Fabricius, and petechial hemorrhages in the skeletal muscles and proventriculus.

## Tissue processing and virus preparation

The collected pooled tissues (bursa of Fabricius, thymus, spleen, and liver) from each flock were processed immediately under aseptic conditions. Approximately 1 g of pooled tissues was finely minced and homogenized in sterile phosphate-buffered saline (PBS, pH 7.2) to prepare a 20% (w/v) suspension. To maintain viral stability and prevent bacterial or fungal contamination, the suspension was supplemented with an antibiotic–antimycotic mixture containing 100 U/mL penicillin, 100 µg/mL streptomycin, and 0.25 µg/mL amphotericin B. The homogenates were subjected to three freeze–thaw cycles to enhance cell lysis and maximize viral release. Subsequently, the suspensions were clarified by centrifugation at 4,000 × g for 15 min at 4°C. The supernatants were carefully collected, aliquoted into sterile cryovials, and stored at − 80°C until further use for DNA extraction and molecular characterization [3]. The clarified tissue homogenate was molecularly pre-screened and confirmed to be negative for major avian immunosuppressive viruses, including IBDV, MDV, and ALV, to eliminate the possibility of co-infection.

## Molecular detection and screening of CIAV by real-time PCR (qPCR)

Following the manufacturer’s guidelines, the FAVORGEN Viral Nucleic Acid Extraction Kit (FAVORGEN Biotech Corp., Cat. No. FAVNK002) was employed to extract total viral DNA from 5 mg of each tissue sample. A mechanical homogenizer (PRO 200, Pro Scientific, USA) was used to process the specimens at 4°C prior to the extraction procedure to maintain sample integrity. The purified DNA was eluted in 50 µL of elution buffer and stored at − 20°C until further analysis. Detection and quantification of the CIAV genome were performed using a highly sensitive TaqMan-based real-time PCR assay as previously described by [14]. The qPCR assay utilized a specific set of primers and a probe designed to target a conserved region of the CAV genome. This set included the forward primer CAV1F (5′-CTC AAG CGA CTT CGA CGA AGA-3′), the reverse primer CAV1R (5′-AGC CTC ACA CTA TAC GTA CCG-3′), and a TaqMan probe (CAV-probe: 5′-FAM-ACA GCG GTA TCG TAG ACG AG-TAMRA-3′) labeled with FAM and TAMRA. The qPCR was performed in a total volume of 20 µL per reaction. Each mixture contained 10 µL of 2 × QuantiNova Probe Master Mix (Qiagen, Germany), 0.5 µL of each primer (forward and reverse), 0.25 µL of the specific probe, and 5 µL of template DNA. Amplification was performed using an Applied Biosystems real-time PCR system (USA) under the following cycling conditions: initial denaturation at 95°C for 2 min, followed by 40 cycles of denaturation at 95°C for 10 s and annealing/extension at 60°C for 30 s. Following the initial screening, samples with the lowest Ct values were utilized for further characterization, including full-genome sequencing and analysis of the partial VP2 gene region.

## Conventional PCR and full-genome and partial *VP2* gene amplification

Following the initial screening, 16 representative positive samples, selected based on the lowest cycle threshold (Ct) values, were subjected to conventional PCR for amplification of the full circular genome (n = 5) and the partial *VP2* gene (n = 10). To amplify the full circular genome (approximately 2.3 kb), three sets of specific overlapping primers (F1/R1, F2/R2, and F3/R3) were used according to the methodology described by [3]. In parallel, the partial VP2 gene was amplified using a specific primer pair targeting a conserved region, as previously described by [15]. All primers used in this study are listed in Table 1. PCR amplification was conducted in a final volume of 25 µL, utilizing 12.5 µL of EmeraldAmp® GT PCR Master Mix (Takara Bio, Shiga, Japan). The reaction included 1 µL (10 pmol/µL) of each forward and reverse primer, 5 µL of template DNA, and 5.5 µL of nuclease-free water. Thermal cycling was performed on a T100™ Thermal Cycler (Bio-Rad, USA) with the following parameters: an initial 5-min denaturation at 95°C, followed by 35 cycles of 95°C for 30 s (denaturation), 56°C for 45 s (annealing), and 72°C for 90 s (extension), ending with a 10-min final extension at 72°C. The specificity of PCR products was confirmed by electrophoresis on a 1.5% (w/v) agarose gel. The presence of clear bands at the expected sizes (850, 900, and 750 bp for the overlapping fragments and approximately 600 bp for the partial VP2 gene) indicated successful amplification and high specificity of the primers.

**Table 1:** Primers used for full-genome and partial VP2 amplification of CIAV isolates

Primer Name	Sequence(‘3 → ‘5)	Target Region	Product Size (bp)	Reference
CIAV-F1	-5’CTAAGATCTGCAACTGCGGA-3’	Genomic region-1) (850	850~bp	[3]
CIAV-R1	-5’CCTTGGAAGCGGATAGTCAT-3’			
CIAV-F2	-5’GACCGTCTGAGGATCCACAA-3’	Genomic region-750) (1650	900~bp	
CIAV-R2	-5’GGCAGTGGTTAGCATTCCAA-3’			
CIAV-F3	-5’TCCGAAGGTTAGTGGCACTA-3’	Genomic region-1550) (2300	750~bp	
CIAV-R3	-5’AGGGCTAGGAAGGATACCGT-3’			
VP2-F	5’-GCAGGAAGTCAATCAAGCAG-3’	Partial VP2 Gene	600~bp	[15]
VP2-R	5’-GGCAGTGGTTAGCATTCCAA-3’			

## Purification, sequencing, and phylogenetic analysis of CIAV isolates

The products for the full-genome fragments (850, 900, and 750 bp) and the partial VP2 segments (~ 600 bp) were electrophoresed on 1% low-melting-point agarose gels. Target bands were excised and purified using the QIAquick® Gel Extraction Kit (Cat. No. 28704, Qiagen, Germany) according to the manufacturer’s instructions. Purified amplicons were eluted in 30 µL of elution buffer and stored at -20 °C until sequencing. The 16 representative isolates underwent bi-directional cycle sequencing utilizing an ABI PRISM® 3730XL Analyzer. This process incorporated BigDye™ Terminator Cycle Sequencing Kits and AmpliTaq DNA polymerase within an MJ Research PTC-225 Peltier Thermal Cycler (Applied Biosystems, USA). To achieve optimal sequence coverage and accuracy, the original PCR primers were employed for sequencing. The resulting sequences were initially screened for identity using the NCBI BLAST tool. Subsequently, Lasergene (DNAStar, version 10) and BioEdit v7.2 [16] were used for the assembly, editing, and curation of the raw sequence data. Sequence alignment at both nucleotide and deduced amino acid levels was performed using the Clustal W module [17]. Evolutionary relationships were evaluated by constructing phylogenetic trees using MEGA X software [17]. The neighbor-Joining method was applied in conjunction with the Tamura-Nei substitution model, and branch reliability was validated through 1000 bootstrap replicates. Upon completion of the analysis, the sequences were deposited in the NCBI GenBank database, and unique accession numbers were obtained for each isolate.

## Recombination analysis

To identify putative recombination events in the sequences of the CAV isolates, RDP v.4.97 software was used to assess the presence of such recombination events using the analytical method of BootScan, and 3Seq. A *p*-value (less than or equal to 0.05) was employed to validate the detection of recombination regions. In addition, upon detection of recombination regions, the evolutionary relationships among the identified parental sequences associated with each recombination event were investigated. The identified putative recombination events were subsequently validated and analysed using SimPlot software [18, 19].

## VP1 protein structure modeling

To further characterize the structural features of VP1 protein in the CIAV isolates, a three-dimensional structure (3D) of the VP1 protein with the reference strain of SD24, was generated using the homology-modelling pipeline via the automatic mode of SWISS-MODEL (Swiss Institute of Bioinformatics; https://swissmodel.expasy.org/ accessed on 01 October 2026). Consequently, the resulting structure for current isolates was detected using PyMOL software (Version 1.7.4, LLC; http://www.pymol.org/) [20–22]. Model quality was evaluated using the Global Model Quality Estimation (GMQE) and QMEAN scoring systems provided by SWISS-MODEL. Structural visualization and superimposition analyses were performed using PyMOL version 1.7.4.

## In Vivo pathogenicity assessment of egyptian CIAV strains

To evaluate the virulence of CIAV in previously unstudied regions, the Assiut isolate (GenBank Accession Nos. PX596994 and PX597000) was used for in vivo trials. This represents the first pathogenic assessment of an isolate from Upper Egypt, offering a critical contrast to existing literature, which has concentrated exclusively on viral strains recovered from northern Egyptian provinces. A total of 100 one-day-old Specific-Pathogen-Free (SPF) chicks (Kom Osheem SPF project, Fayoum, Egypt) were randomly divided into two groups (n = 50 per group). All experimental procedures were conducted under strict biosecurity measures within a high-standard, properly controlled isolation facility equipped with strictly monitored negative-pressure isolators. The chicks were housed under hygienic conditions with filtered air supply and restricted personnel access to entirely prevent any horizontal transmission or external contamination. To rigorously confirm the SPF and CIAV-negative status prior to challenge, random meconium and embryonic tissue samples from the same hatch were molecularly screened using the TaqMan quantitative qPCR assay. Furthermore, serological testing using a commercial ELISA kit (IDEXX Laboratories, Inc, USA) verified the absence of maternally derived anti-CIAV antibodies, ensuring zero baseline contamination. Birds in the challenged group were administered an intramuscular (I/M) injection of 0.1 mL, containing 4 × 10^3^ viral DNA copies. Conversely, the control group was injected with an identical volume of sterile phosphate-buffered saline (PBS). Birds were monitored daily for 35 days post-infection for clinical signs and mortality. Daily clinical scoring was performed based on a standardized 4-point scale evaluating depression, feather ruffling, and paleness of the comb/wattle: Grade 0 (Normal); Grade 1 (Mild depression and slight feather ruffling); Grade 2 (Moderate depression, visible paleness, and reluctance to move); and Grade 3 (Severe depression, severe paleness, or death). Individual body weights (BWs) were recorded weekly (7, 14, 21, 28, and 35 dpi). To assess lymphoid atrophy and growth retardation, three chickens from each group were randomly euthanized at each of the five weekly time points (totaling 15 birds per group for destructive sampling). The remaining 35 birds per group served as the baseline population to ensure a robust and statistically sound sample size (n = 15 remaining birds at the final timelines) for computing longitudinal parameters like weekly Packed Cell Volume (PCV%), antibody kinetics, and cumulative mortality rates while strictly balancing animal welfare and ethical constraints. The relative organ-to-body weight ratios (organ index) for the thymus, spleen, and bursa of Fabricius were calculated using the standard formula according to [23]. Heparinized blood samples were collected weekly to determine packed cell volume (PCV%) using the microhematocrit method and hemoglobin (Hb) levels. Birds with PCV < 25% were considered anemic, according to [24]. Serum specimens were analyzed for the presence of anti-CIAV antibodies utilizing the BioChek CIAV Antibody ELISA kit (BioChek B.V., Netherlands) according to the manufacturer’s protocols [25].

Viral load dynamics in lymphoid organs, bone marrow, and the liver were tracked using qPCR, with results expressed in terms of mean log10 DNA copies/g [26]. Representative tissues (the thymus, bone marrow, and spleen) were fixed in 10% neutral buffered formalin, processed routinely, and stained with hematoxylin and eosin (H&E). Sections were examined microscopically for characteristic lesions, including lymphoid depletion and bone marrow aplasia [1].

## Standard curve-based absolute qPCR for viral DNA

To evaluate viral load dynamics and tissue tropism during the pathogenicity trial, absolute CIAV DNA quantification was performed on the liver and lymphoid tissues (spleen, thymus, and bone marrow), according to [26]. Total DNA was isolated from 5 mg of each tissue specimen. The AriaMx qPCR System (Agilent Technologies, USA) was utilized for quantification. Absolute quantification was achieved using a standard curve generated from ten-fold serial dilutions of a certified CIAV vaccine with a known genomic concentration. The resulting standard curve demonstrated high linearity (R^2^ = 0.994) and a slope of -3.077, yielding an amplification efficiency of 111.33% and a y-intercept of 12.81. The assay exhibited high diagnostic sensitivity, with a limit of detection (LoD) of 10^1^ viral DNA copies per reaction and a limit of quantification (LoQ) of 10^2^ viral DNA copies per reaction. To maintain analytical standardization across varying samples, the final viral concentrations were normalized and expressed as log_10_ DNA copies per milligram of tissue (log_10_ copies/mg tissue), calculated based on the initial tissue weight (5 mg) utilized for total DNA extraction. All reactions were performed in triplicate, and viral concentrations were reported as log10 DNA copies per µL of template DNA. All samples were concurrently tested for other major avian pathogens using qPCR protocols [27–41] (Table 2).

**Table 2:** Oligonucleotide primers and TaqMan probes utilized for the differential diagnosis and exclusion of extraneous avian viral and bacterial pathogens

ID	Primer and probe sequences	Reference
Avian influenza virus-M-gene	sep1:AGATGAGTCTTCTAACCGAGGTCG sep2:TGCAAAAACATCTTCAAGTCTCTG sep-probe: FAM-TCAGGCCCCCTCAAAGCCGA-TAMRA	[26]
Avian influenza virus-H5 subtype	H5LH1: ACATATGACTACCCACARTATTCAG H5RH1:AGACCAGCTAYCATGATTGC H5PRO: FAM-TCWACAGTGGCGAGTTCCCTAGCA-TAMRA	[27, 28]
Newcastle disease virus	F + 4839: TCCGGAGGATACAAGGGTCT F -4939: AGCTGTTGCAACCCCAAG F + 4894: FAM-AAGCGTTTCTGTCTCCTTCCTCCA-TAMRA	[29]
Infectious bursal disease virus	F/AUS GU: TCACCGTCCTCAGCTTACCCACATC R/AUS GL: GGATTTGGGATCAGCTCGAAGTTGC	[30]
Marek’s disease virus	MDV-F: GGCACGGTACAGGTGTAAAGAG MDV-R: GCATAGACGATGTGCTGCTGAG	[31]
Fowl adenovirus	F- 5ʹ-ACATGGGAGCGACCTACTTCGACA-3ʹ R- 5ʹ-TCGGCGAGCATGTACTGGTAAC-3ʹ	[32]
Avian Encephalomyelitis virus	-F:5’GAATTAGCTCCTGGTAAACCTCG-3’ R: 5’-CTCTATCGCAACACCCTCAGG-3’	[33]
Chicken astrovirus	Fast-deg-2 5’- GCA TGG CTC CAC CGT AAG C -3’ Rast-2 5’- ACA CTC CCA GCA ACA TTT G -3’	[34]
E. coli phoA	F- 5ʹ-CGATTCTGGAAATGGCAAAAG-3ʹ R- 5ʹ-CGTGATCAGCGGTGACTATGAC	[35]
Salmonella invA	F- 5ʹ-GTGAAATTATCGCCACGTTCGGGCAA-3ʹ R- 5ʹ-TCATCGCACCGTCAAAGGAACC-3ʹ	[36]
Clostridia peregrines Net-B toxin gene	F- 5ʹ-GCTGGTGCTGGAATAAATGC-3ʹ R- 5ʹ-TCGCCATTGAGTAGTTTCCC-3ʹ	[37]
Mycoplasma MSv gene	F: 5’-GGC CAT TGC TCC TRC TGT TAT-3’ R: 5’-AGT AAC CGA TCC GCT TAA TGC-3’	[38]
Staphylococcus aureus (nuc) gene	F: 5’GCGATTGATGGTGATACGGT-3’ R: 5’AGCCAAGCCTTGACGAACTAAAGC-3’	[39]
Streptococcus spp. pbp1A gene	F: 5’AAACAAGGTCGGACTCAACC-3’ R: 5’AGGTGCTACAAATTGAGAGG-3’	[40]

## Results

### Clinical and macroscopic findings

Clinical surveillance conducted across ten Egyptian governorates (2024–2025) identified a disease profile closely linked to host age. Acute Chicken Infectious Anemia (CIA) was the primary finding in young broilers (2–3 weeks old), manifesting as profound depression, growth retardation, and characteristic pallor of the skin and combs. Mortality in these cohorts varied between 3 and 10%, whereas infections in older breeder and layer flocks (22–27 weeks) remained largely subclinical. A post-mortem examination of 400 specimens revealed systemic lesions, characterized by marked atrophy of the bursa of Fabricius and thymus. The bone marrow exhibited a distinct yellowish-white discoloration, signifying hematopoietic failure. Furthermore, petechial hemorrhages in the proventriculus and ecchymoses on the wings (blue wing disease) were prevalent, often accompanied by hepatomegaly and splenomegaly.

## Molecular screening and DNA amplification

Among the 400 tissue specimens collected, 120 (30%) were identified as positive for CIAV DNA via TaqMan-based qPCR screening. From these positive cases, sixteen representative isolates were randomly selected for further genetic characterization via conventional PCR. These included five isolates for full-genome amplification (~ 2.3 kb) and ten (10) isolates for partial *VP2* gene amplification (~ 600 bp). Notably, agarose gel electrophoresis confirmed the successful amplification of all 16 isolates, yielding clear bands at the expected molecular sizes with high primer specificity. No amplification was detected in the negative controls. The resulting high-quality amplicons were purified and subjected to bi-directional sequencing. This was performed to characterize their molecular profiles and evaluate the evolutionary dynamics of the circulating strains.

## Sequence and phylogenetic analysis

**Fig. 2:**
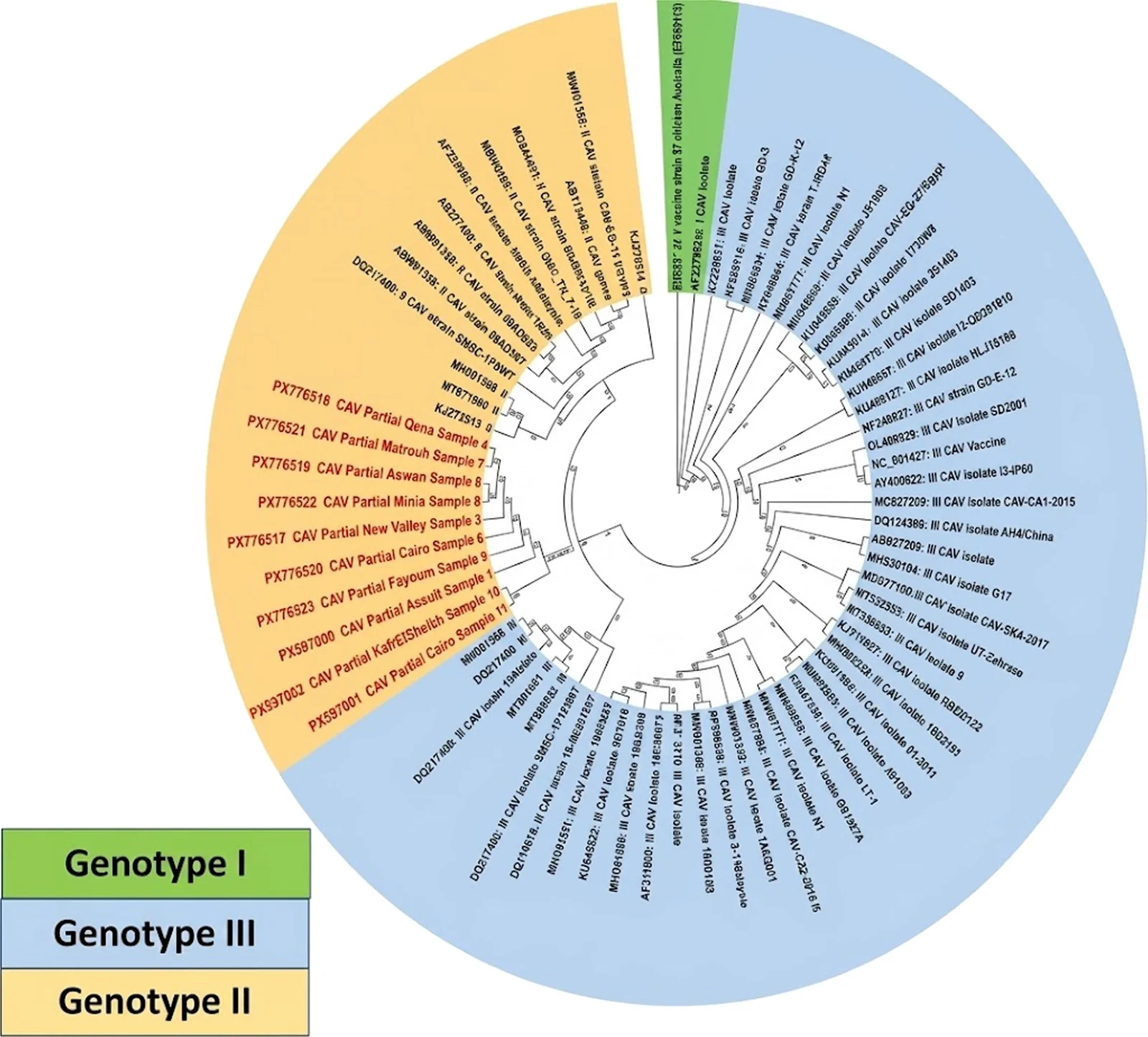
A phylogenetic tree based on partial *VP2* gene sequencing of the studied CIAV isolates in comparison to reference and Egyptian CIAV strains retrieved from GenBank. Phylogenetic analysis of all ten CIAV isolates exhibited clustering in genotype II (highlighted in red). The tree was generated using the Maximum Likelihood approach with 1,000 bootstrap replicates in MEGA (version 7), using the general likelihood reversible substitution model

**Fig. 3:**
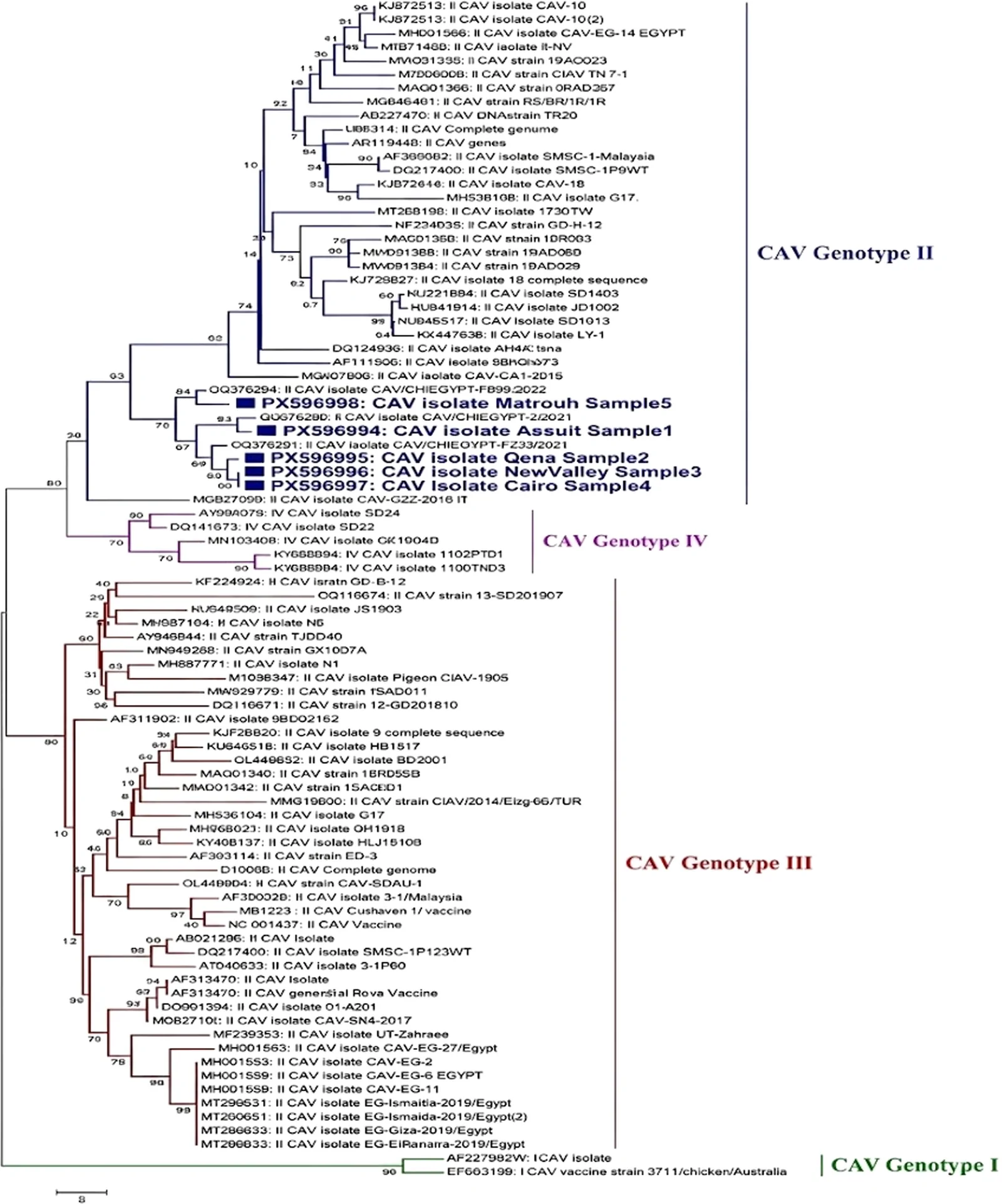
A collective phylogenetic tree based on full genome CIAV sequencing was constructed using nucleotide sequence alignments in comparison to other reference strains from different geographical regions retrieved from GenBank*.* The phylogenetic analysis revealed that 5 Egyptian CIAV isolates (indicated by blue squares and bold font) grouped within genotype II, showing close genetic relationships with previously reported Egyptian and international genotype II strains. Sequence alignment was performed using ClustalW, and the phylogenetic tree was designed using the Maximum Likelihood approach with 1,000 bootstrap replicates in MEGA (version 7)

Phylogenetic analysis was conducted among different CIAV strains constructed on the partial *VP2* gene sequences retrieved from the GenBank database. Based on partial sequencing, the construction of the phylogenetic tree demonstrated that ten Egyptian CIAV isolates clustered tightly within genotype II, indicating a high degree of genetic relatedness among Egyptian circulating strains. (Fig. 2). To validate this classification, full genome sequencing and a phylogenetic tree of the CIAV from five representative isolates, namely PX596994: CAVAssuit/sample1, PX596995: CAVQena/sample2, PX596996: CAVNewValley/sample3, PX596997: CAVCairo/sample4, and PX596998: CAVMatrouh/sample5, belonged to CIAV genotype II. These five sequences were subsequently deposited to the GenBank database with their accession numbers PX596994–PX596998 (Figs. 2 and 3). Comparative sequence analysis of five current sequences was aligned with reference sequences from various CIAV genotypes and vaccines retrieved from GenBank. Interestingly, our isolates, including PX596994: CAVAssuit/sample1, PX596995: CAVQena/sample2, PX596996: CAVNewValley/sample3, PX596997: CAVCairo/sample4, and PX596998: CAVMatrouh/sample5, shared the highest genetic similarity with previously reported genotype II strains, particularly MG827098: II CAV/2015/Egypt and MG827099: II CAV/GZ2-2016/Egypt (Egyptian strains isolated in 2015–2016), exhibiting nucleotide and amino acid similarity ranging from 97 to 98%. At the global scale, the five contemporary isolates demonstrated close genetic relatedness to genotype II reference strains circulating in multiple regions, including China (DQ124936: II CAV/AH4, KU645517: II CAV/SD1513), Australia (U65414: II CAV/704), Japan (AB027470: II CAV/TR20), Malaysia (DQ217400: II CAV/SMSC-1P9WT), USA (AF311900: II CAV/98D06073), and Brazil (MG846491: II CAV/RS/Brazil/15/1R), with nucleotide and amino acid identities between 96 and 98% (Figs. 2 and 3). In contrast, slightly lower similarity levels (85%–97%) of the recorded isolates were observed when compared with CIAV vaccine-related strains, including D10068: III CAV 26P4/Netherland-2007, AF313470: III CAV/Del Rose, M81223: III CAV Cuxhaven1, NC_001427: III CAV, and EF683159: I CAV vaccine 3711/Australia (genotype I). Likewise, the analyzed five isolates also demonstrated a relatively high nucleotide and amino acid identity (95–98%) to genotype III Egyptian strains, such as MG827100: III CAV/SK4-2017/Egypt, MH001555: III CAV/6/2017EGYPT, MT268631: III CAV/Ismailia/2019/Egypt, and MT268633: III CAV/ElBeheira/2019/Egypt, respectively (Table S2, supplementary materials). Importantly, pairwise comparison among the five newly sequenced isolates revealed 99% nucleotide and amino acid similarity to each other, indicating a very close genetic relationship and likely similar origin. Overall, these results demonstrate that our Egyptian strains circulating in various Egyptian governorates are phylogenetically related to local and global strains, reflecting ongoing viral circulation and genetic exchange across geographical regions.

## Recombination analysis

**Fig. 4:**
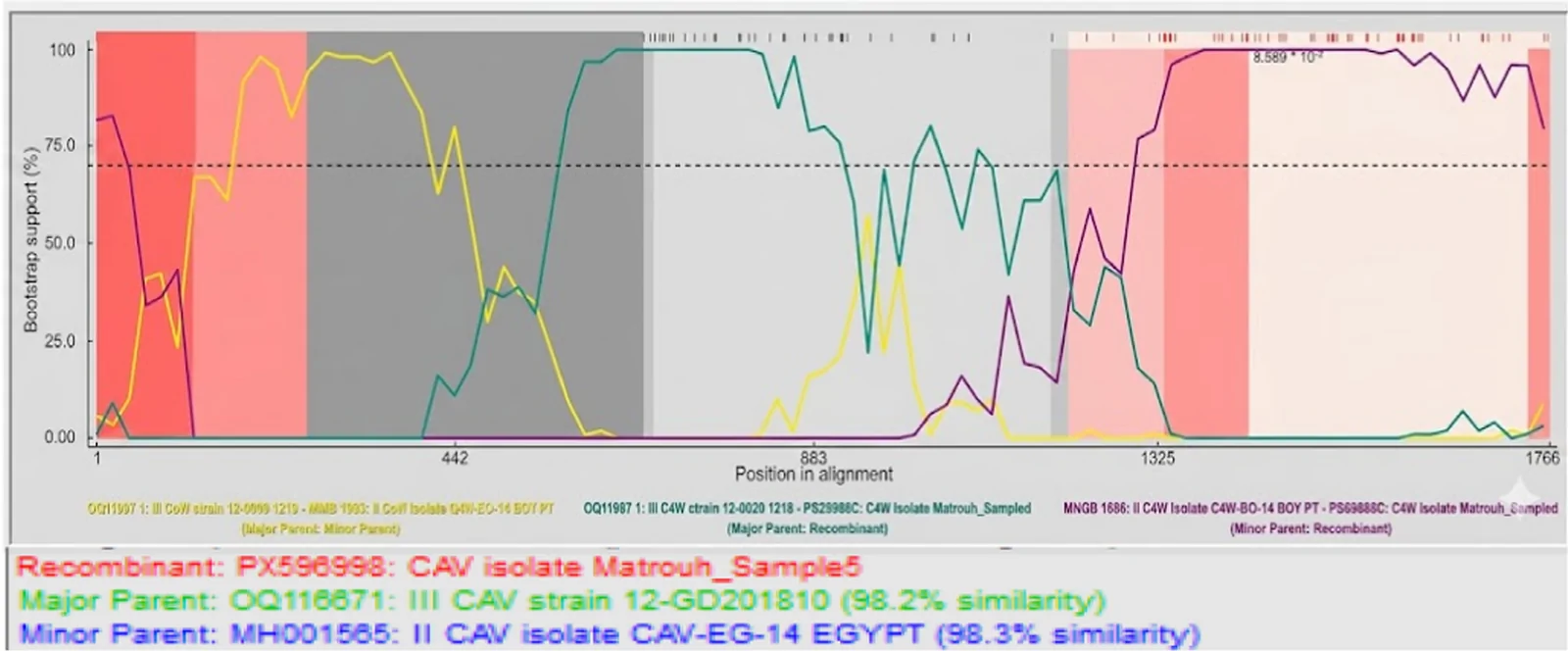
Recombination analysis of the Egyptian isolate PX596998: CAVMatrouh/ sample5 performed using RDP4 software. The recombinant region is indicated in pale pink. Potential parents involved in the recombination events as follows: the OQ116671: III CAV strain 12-GD201810 of genotype III Chinese isolate is a major parent. MH001565: II CAV isolate CAV-EG-14 EGYPT of genotype II Egyptian isolate is a minor parent

Recombination detection analysis using RDP 4.0 software was performed, which indicated that PX596998: CAVMatrouh/sample5 was a potential recombinant strain involved in a robust recombination event. This recombination event was supported by algorithm within the RDP4 suite of BootScan (P value = 8.58 × 10^–2^). The recombinant CIAV isolate originated from an inter-genotypic recombination between two parental strains: the major parent was identified as OQ116671: III CAV strain 12-GD201810 of genotype III, whereas the minor parent was MH001565: II CAV isolate CAV-EG-14 EGYPT of genotype II (Fig. 4). The predicted recombination breakpoint analysis indicated that the primary recombinant fragment spanned nucleotide positions between 1214 (95% confidence interval: 1194–1432) and 257 (95% confidence interval: 1740–682), whereas the complementary genomic regions extended from 1740 to 682 within the circular genome.

## Mutation and homology modeling analyses

A comparative analysis of amino acid sequence (~ 588 amino acids “aa”) demonstrated that our Egyptian CIAV isolates (n = 5) exhibited a significant degree of similarity with previously reported Egyptian genotype II strains, especially in comparison to the reference strain (CAV/2015/Egypt; MG827098). These sequences showed conserved regions with different amino acid substitutions in the relevant regions. In the N-terminal region (aa 1–130), all isolates exhibited a singular alanine (A) substitution at position 16, a change also noted in multiple global reference strains. In the hypervariable region of VP1 (aa 116–126) (Figure S1, Supplementary Materials), isolates PX596996-PX596998 displayed a serine (S) substitution, and PX596998 also demonstrated a leucine (L) substitution at position 126. In the central region (aa 220–240), several mutations were identified, such as isoleucine (I) substitutions in PX596994 and PX596998.

Also, variations were found in the motif that spans positions 235–245. These changes include proline (P), serine (S), leucine (L), and histidine/glycine (H/G) [P/S/L/HG pattern], which is a sign of genotype III strains among all isolates. All five isolates had a substitution in methionine (M) at position 254, which is what was found in previously documented Egyptian strains. Moreover, PX596998 displayed a distinctive (L) substitution at position 283. Three different mutations were identified in the central region of VP1 (aa 250–320) (Figure S1, Supplementary Materials), All Egyptian field isolates showed a substitution to lysine (K) at position 296 compared to the reference sequence. At position 301, a substitution to glutamic acid (E) was observed. The isolates also had a substitution to methionine (M) at position 306. In the VP2 region at aa 467, a cysteine (C) substitution was currently detected in PX596998, while the other isolates showed no changes. Importantly, the recorded isolates did not show any amino acid substitutions in the hypervariable region of VP1 (aa 139–151). In the C-terminal region, all isolates also showed an isoleucine (I) substitution at position 533.

**Fig. 5:**
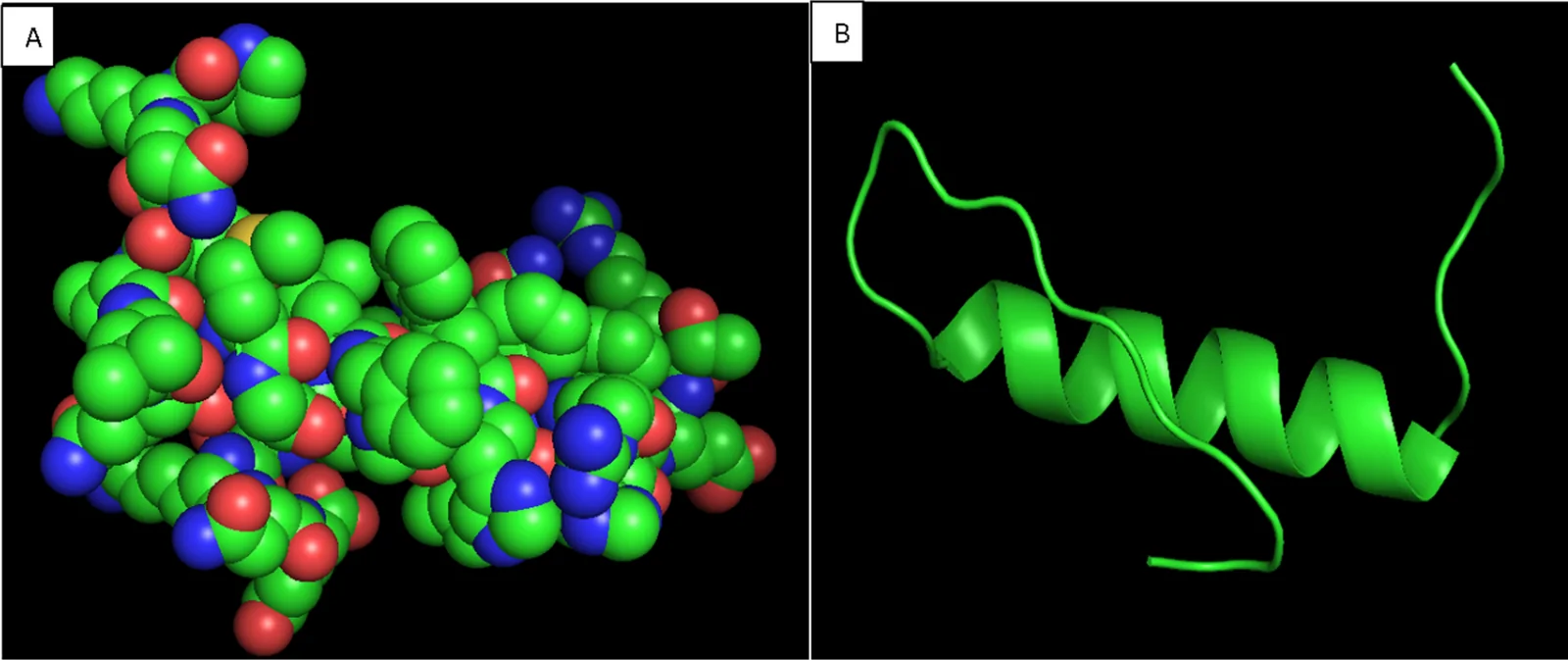
Tertiary structure Modeling of VP1 protein in CIAV isolates of Genotype II. The sphere (**A**) and carton (**B**) representations of the VP1 protein

**Fig. 6:**
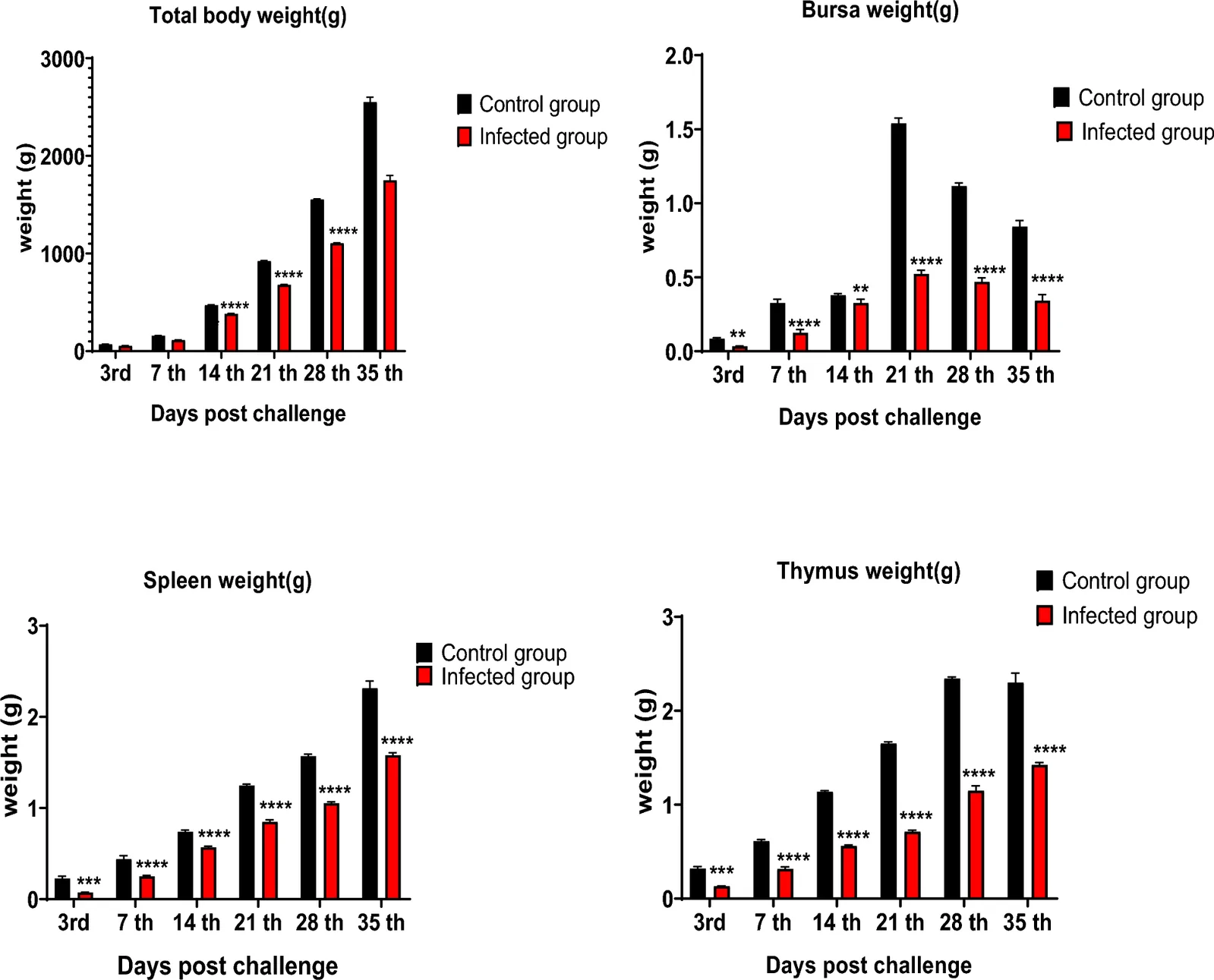
Dynamic changes in growth performance and lymphoid organ development. The bar sequences illustrate the progressive impact of experimental infection with the Assiut CIAV Genotype II isolate on (**A**) Total body weight, (**B**) Bursa of Fabricius weight, (**C**) Spleen weight, and (**D**) Thymus weight over a 35-day observation period. Data are presented as Mean ± SEM. Asterisks indicate significant differences between the infected group (red bars) and the control group (black bars) as determined by Two-way ANOVA followed by Sidak’s multiple comparisons test (*P < 0.05, **P < 0.01, ***P < 0.001, ****P < 0.0001). Note the severe and early-onset atrophy in the thymus and bursa starting from 3 days post-challenge

To further characterize the recent CIAV isolates, a predicted three-dimensional (3D) structure was generated using homology modeling method using the SWISS-MODEL automatic mode. The resulting model was analyzed and visualized using Chimera software to detect the target protein characteristics. Using PyMOL (Version 1.7.4, LLC, accessed on 6 January 2026), we analyzed the VP1 protein structure of the Upper Egyptian CIAV Genotype II isolate with a GMQE value of 0.75, and a QMEANDisCo global score of 0.76 ± 0.05. The protein was tightly packed, as expected for a major structural capsid protein. The sphere and cartoon representations (Fig. 5 A, B) illustrated a densely globular structure, indicative of a stable structural unit forming the icosahedral capsid, featuring a dominant alpha-helical segment interspersed with highly flexible loop regions around it. The stereochemical and structural validity of the generated VP1 3D model was rigorously verified using standard quality metrics. Ramachandran plot analysis demonstrated that 96.88% of the amino acid residues were located within the most favored regions, confirming excellent geometric quality Fig.6.

## In Vivo pathogenicity of upper egyptian CIAV genotype II

### Impact on body weight and lymphoid organs development

**Fig. 7:**
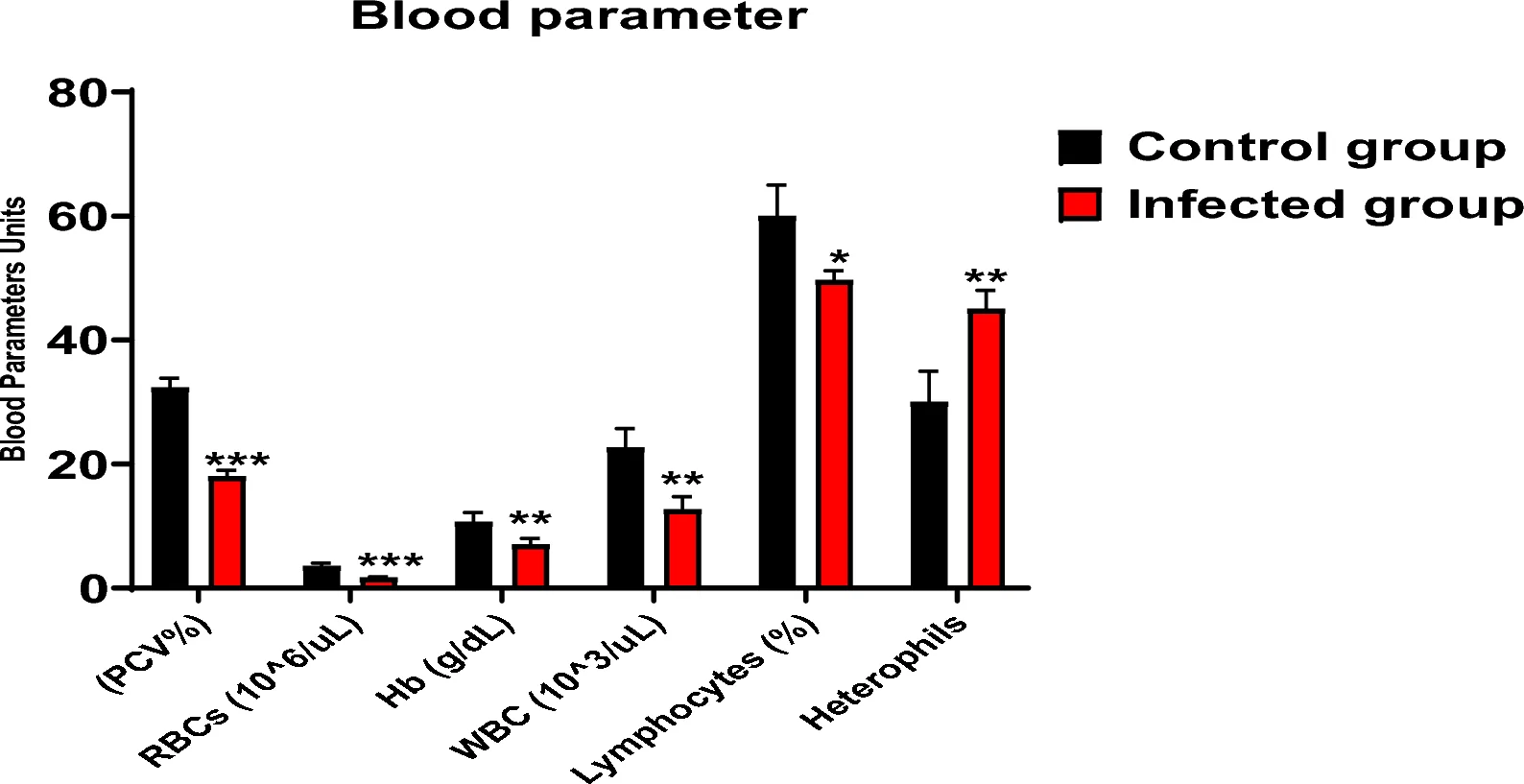
Hematological profile at 14 days post-infection (dpi). Comparative bar chart displaying the impact of the Assiut CIAV Genotype II isolate on key blood parameters. A significant reduction (Anemia and Leukopenia) is evident in Packed Cell Volume (PCV%), total Erythrocyte count (RBCs), Hemoglobin (Hb), and total Leukocyte count (WBC). Differential counts show a significant decline in Lymphocytes (Lymphopenia) accompanied by a compensatory increase in Heterophils (Heterophilia). Statistical significance is indicated by asterisks (*P < 0.05, **P < 0.01, ***P < 0.001) based on an unpaired t-test comparing the infected group (red) to the control group (black)

Experimental infection with the Assiut genotype II isolate induced a significant growth retardation and progressive atrophy of primary and secondary lymphoid organs (Table S3, supplementary materials; Fig. 7). A marked decline in total body weight was recorded beginning at 14 days post-infection (dpi), indicating a significant impact of the virus on bird development (383 ± 2.08 g vs. 471.7 ± 1.67 g in control; P < 0.01), reaching its peak at 35 dpi (P < 0.0001). The most dramatic impact was recorded in the thymus, where a sharp decline in weight was evident as early as 3 dpi (0.13 ± 0.00 g) compared to the control (0.32 ± 0.01 g), maintaining a highly significant atrophy throughout the 35-day period (P < 0.0001). Similarly, the Bursa of Fabricius and Spleen weights showed consistent and significant reductions, reflecting a state of severe and prolonged immunodepletion induced by the Upper Egyptian strain.

### Hematological profile and bone marrow suppression

The hematological analysis at the peak of infection revealed severe pancytopenia (Table S4, supplementary materials; Fig. 7). The infected group exhibited a profound drop in hematocrit (PCV%) to 18 ± 0.58%, falling significantly below the normal threshold (> 25%) and the control group (32.33 ± 0.88%; P < 0.001). This was accompanied by a significant reduction in RBC count (1.67 ± 0.09 × 10⁶/µL) and hemoglobin levels (7 ± 0.58 g/dL). Furthermore, a significant leukopenia and lymphopenia were recorded, alongside a compensatory increase in heterophils (45.67 ± 1.73%), confirming the virus’s destructive effect on the hematopoietic cells in the bone marrow.

### Serological response and antibody kinetics

**Fig. 8:**
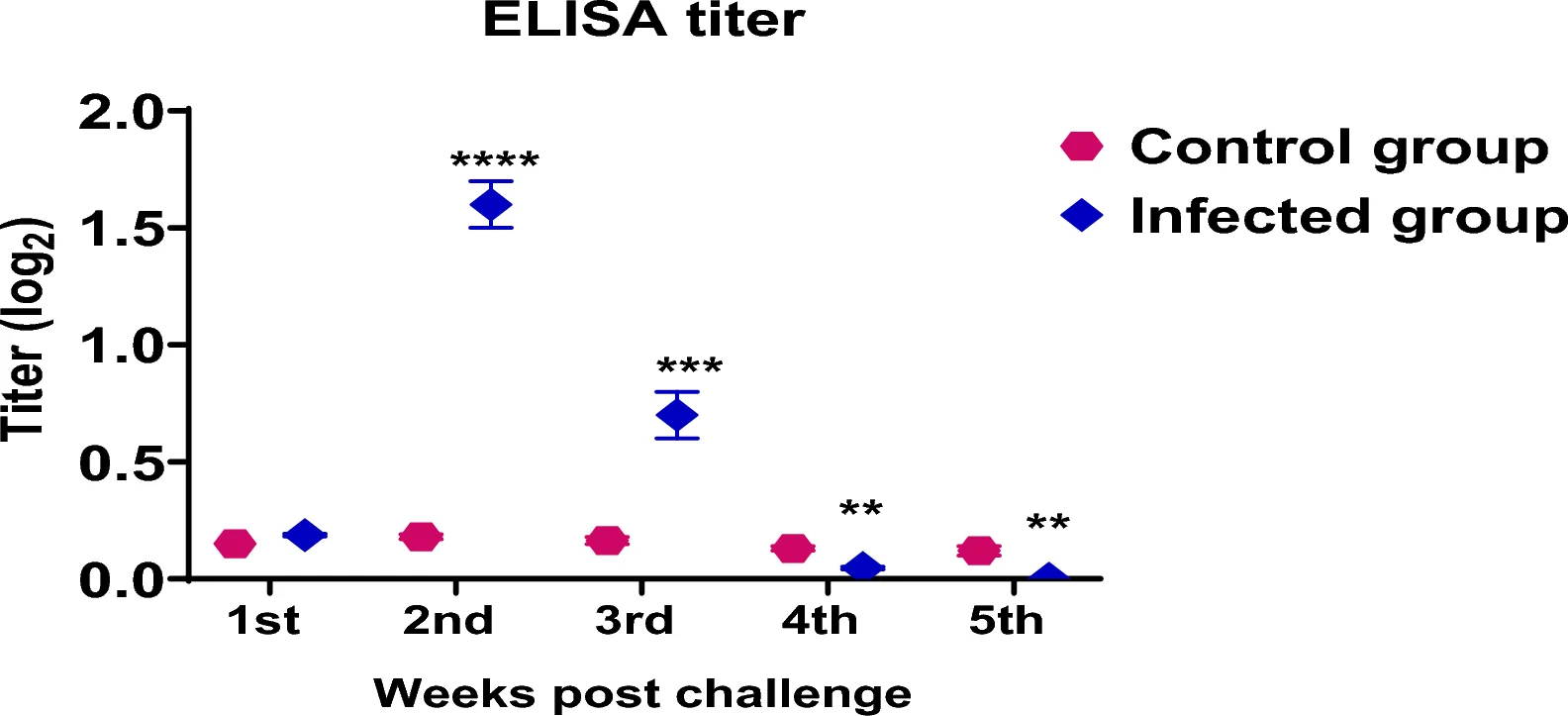
Kinetics of humoral immune response (ELISA titers). The longitudinal profile illustrates the anti-CIAV antibody levels (log_2_ S/P ratio) in SPF chickens over 5 weeks post-challenge. The infected group (blue diamonds) exhibited a significant but transient seroconversion peaking at the 2nd week, followed by a precipitous decline to undetectable levels by the 5th week. The control group (pink hexagons) remained consistently negative. Significant differences are indicated by asterisks (**P < 0.01, *** P < 0.001, **** P < 0.0001) based on Two-way ANOVA

Anti-CIAV antibody titers, measured as S/P ratios, showed a transient peak at 2 weeks post-infection (wpi) (1.60 ± 0.058), significantly higher than the control group (P < 0.0001) (Table S5, supplementary materials; Fig. 8). However, a rapid and sharp decline in antibody levels was observed by 4 wpi, eventually falling below the detection limit by 5 wpi (0.00 ± 0.00). This rapid decline reflects severe immunodepletion and the exhaustion of the B-cell population, rendering the birds unable to maintain a sustained humoral immune response.

### Dynamics of viral load and tissue distribution

**Fig. 9:**
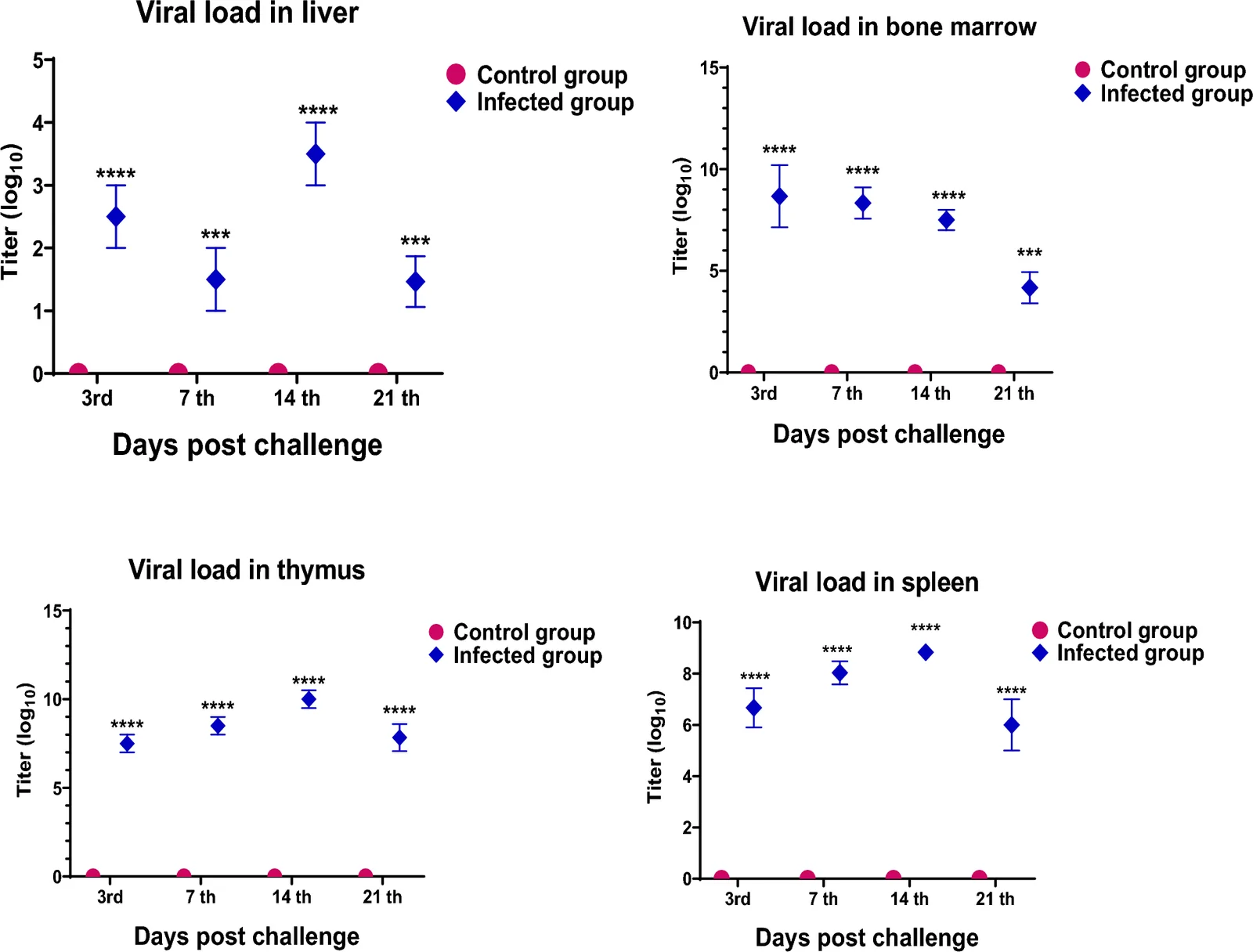
Quantitative dynamic profiling of CIAV viral loads in various tissues. Real-time qPCR kinetics showing the viral DNA concentration (log_10_ copies/mg) in the (**A**) Liver, (**B**) Bone marrow, (**C**) Thymus, and (**D**) Spleen of SPF chickens post-challenge. The infected group (blue diamonds) exhibited high and sustained viral replication across all tissues compared to the negative control group (pink hexagons). Note the peak viral replication in the thymus reaching 10 log_10_ at 14 dpi. Data are presented as Mean ± SEM. Asterisks indicate high statistical significance (***P < 0.001, **** P < 0.0001) based on Two-way ANOVA

**Fig. 10:**
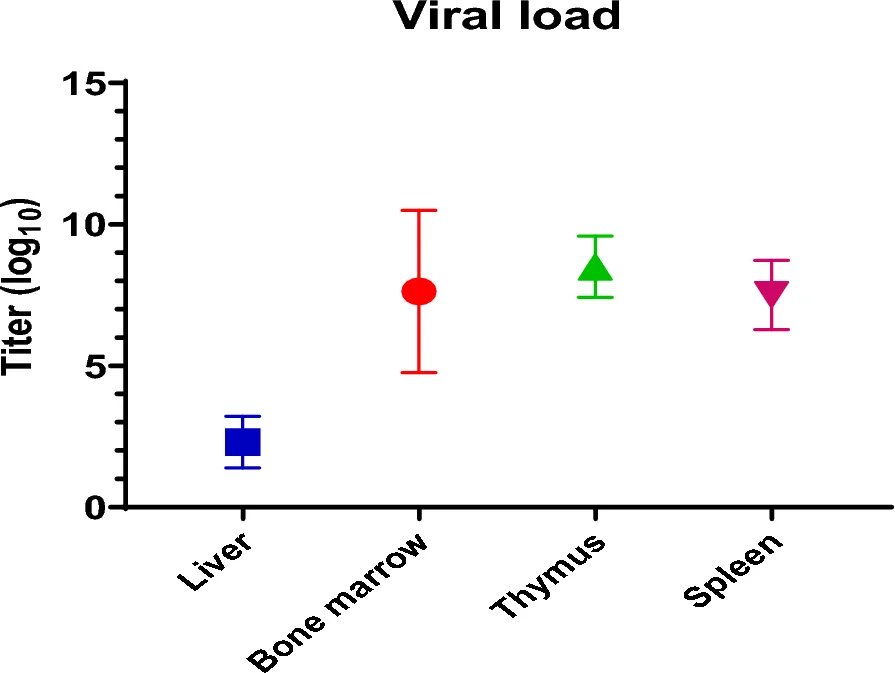
Comparative tissue tropism and mean viral load distribution. The scatter plot represents the mean CIAV viral titers (log_10_ copies/mg) across different organs (Liver, Bone marrow, Thymus, and Spleen) in the infected group throughout the study period. Each symbol represents the mean value, and error bars represent the SD. The plot highlights the high viral affinity for hematopoietic and lymphoid tissues (Bone marrow and Thymus) compared to the liver, emphasizing the organ-specific replication strategy of the Assiut Genotype II isolate

The viral load dynamics quantified by qPCR (Table S6, supplementary materials; Figs. 9 and 10) demonstrated a rapid systemic dissemination of the Assiut isolate. The thymus acted as the primary site of early replication, reaching its peak viral load at 7 dpi (9.50 ± 0.25 log₁₀ copies/mg). Interestingly, the bone marrow showed the highest viral persistence, with loads peaking at 14 dpi (9.00 ± 0.22 log₁₀ copies/mg) and remaining high until 21 dpi. The spleen and liver also harbored significant viral loads, peaking at 14 dpi (6.40 ± 0.18 and 5.20 ± 0.15 log₁₀ copies/mg, respectively), confirming the multi-organ tropism and high replication efficiency of the circulating genotype II strains in Upper Egypt. Rigorous diagnostic exclusion was performed to rule out potential co-infections; all samples tested negative for the list of differential bacterial and viral agents.

### Gross pathological lesions (In Vivo Trial)

Experimental infection with the Assiut Genotype II isolate induced characteristic pathognomonic lesions in SPF chicks (Figure S2, supplementary materials). The most prominent clinical hallmark was "blue-wing disease," presenting as severe cyanosis and subcutaneous hemorrhages on the wing tips. Systemic hemorrhages were consistently observed, including extensive petechiae and ecchymoses in the breast and thigh muscles, as well as severe mucosal hemorrhages at the proventriculus-gizzard junction. The post-mortem examination further revealed a profound impact on the hematopoietic and lymphoid systems. The bone marrow in the femurs was pale and fatty, replacing the normal deep red hematopoietic tissue. Severe atrophy of the lymphoid organs was a defining feature, with the thymus reduced to translucent remnants and marked involution of the Bursa of Fabricius. Additionally, the kidneys were enlarged and pale with visible urate deposition (nephrosis) in several acute cases.

### Histopathological examination

**Fig. 11:**
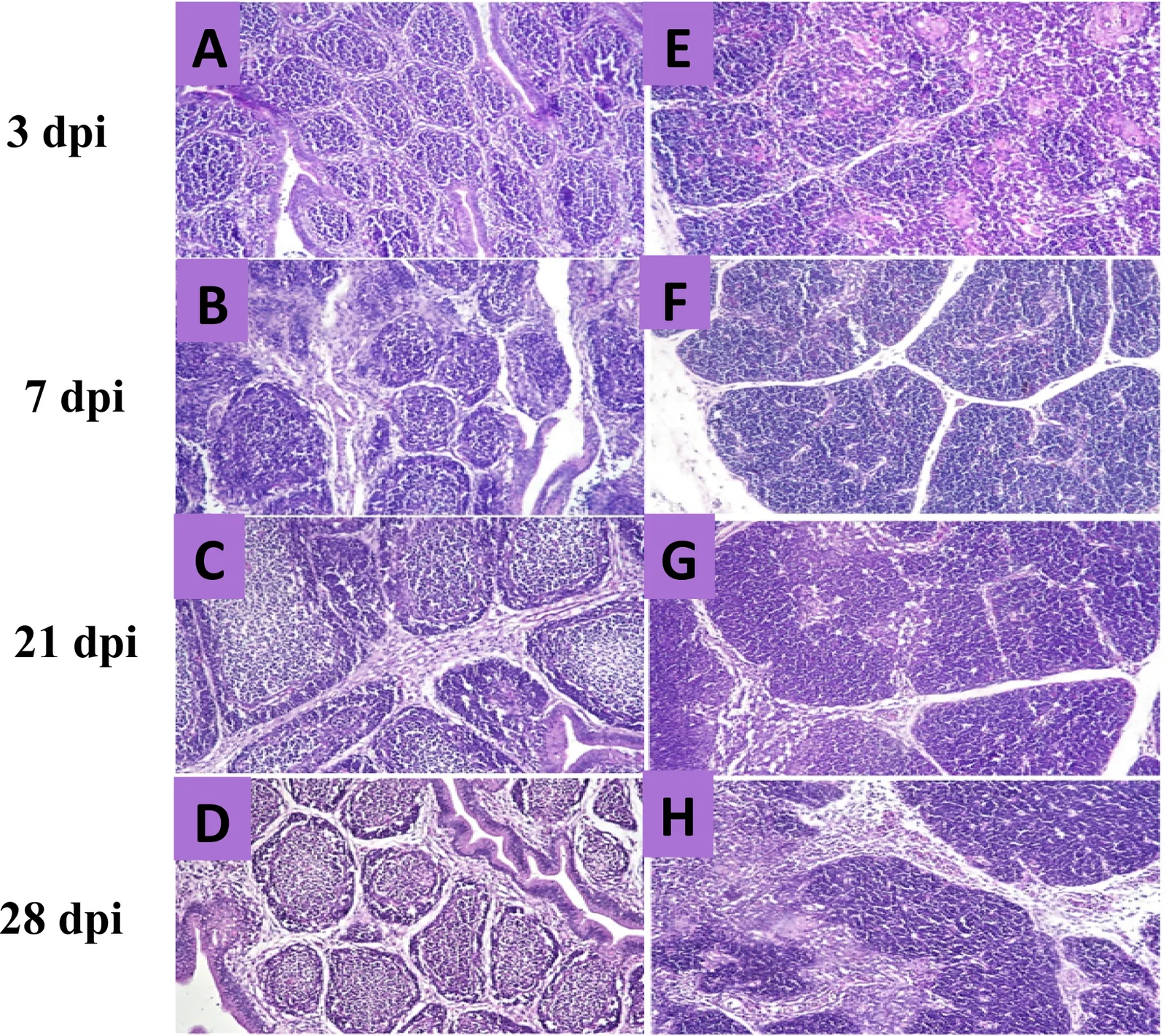
Histopathological progression of CIAV-induced lesions in primary lymphoid organs. In the Bursa of Fabricius, lesions progressed from early edema (**A**) and acute fibrosis/atrophy at 7 dpi (**B**) to chronic diffuse lymphoid depletion by 28 dpi (**D**). In the thymus, early necrotic foci and heterophilic infiltration at 3 dpi (**E**) evolved into severe, extensive lymphocytolysis and depletion by the end of the trial (**H**). (**F**) and (**G**) illustrate the intermediate stages of moderate inflammatory response and lymphoid reduction (H&E stain, 100 × ; Scale bar = 100 µm)

**Fig. 12:**
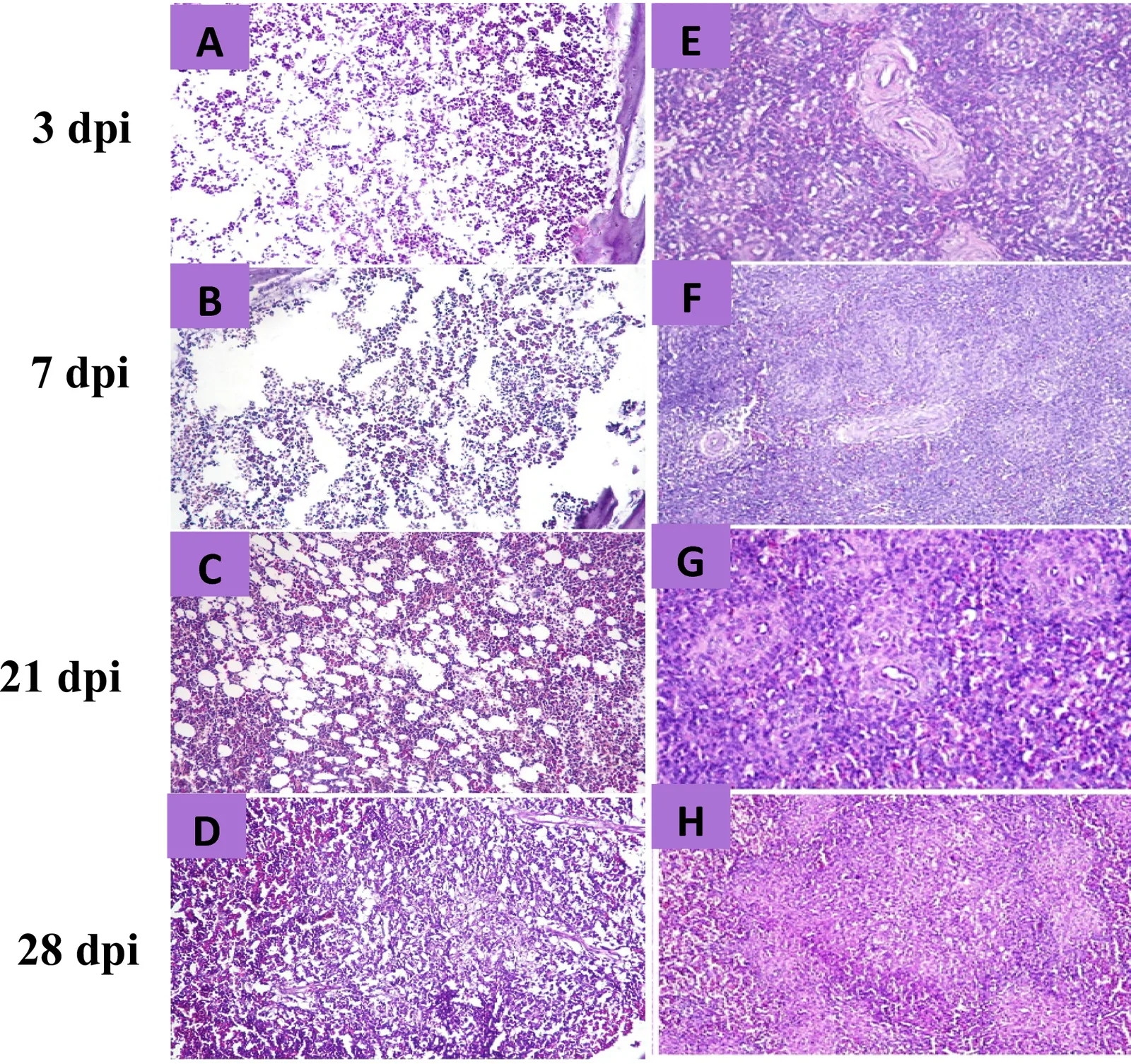
Histopathological progression in the bone marrow and spleen of CIAV-infected chickens. Bone Marrow: (**A**) 3 dpi: moderate hematopoietic and erythrocytic depletion with replacement by interstitial edema and loose connective tissue. (**B**) 7 dpi: presence of focal cellularity indicating a transient reduction in depletion severity. (**C**) 21 dpi: profound marrow atrophy with extensive replacement of hematopoietic elements by adipose and connective tissues. (**D**) 28 dpi: persistent erythropoietic suppression accompanied by mild focal fibrosis. Spleen: (**E**) 3 dpi: marked reticular cell hyperplasia alongside significant depletion of the white pulp. (**F**) 7 dpi: progressive loss of lymphoid elements within the white pulp. (**G**–**H**) 21–28 dpi: advanced chronic lymphoid exhaustion characterized by severe and diffuse white pulp depletion (H&E stain, 200 × ; Scale bar = 200 µm)

Microscopic evaluation of the Bursa of Fabricius in the challenged group demonstrated early-stage inflammatory changes by 3 days post-infection (dpi). These alterations were primarily characterized by interstitial edema and significant infiltration of inflammatory leukocytes. By 7 dpi, progressive damage was evident through severe interstitial fibrosis and a marked reduction in the size of lymphoid follicles. The chronicity of infection led to severe, diffuse lymphoid depletion and lymphocytolysis by 21 and 28 dpi, with prominent heterophil infiltration within the follicles. Similarly, the thymus exhibited moderate lymphocytolysis and necrotic foci as early as 3 dpi. These lesions progressed to severe lymphoid depletion and extensive lymphocytolysis by the end of the experiment (28 dpi), reflecting a state of severe lymphoid exhaustion (Fig. 11). Histological analysis of the bone marrow from infected chickens demonstrated a profound decline in hematopoietic cellularity. By 3 days post-infection (dpi), a moderate depletion of erythroid and myeloid precursor cells was evident, with the hematopoietic space being progressively replaced by loose connective tissue. Although a transient focal recovery was observed at 7 dpi, severe depletion recurred by 21 dpi, with hematopoietic tissue being largely replaced by adipocytes and connective tissue. In the spleen, early infection (3 dpi) induced severe hyperplasia of reticular cells. However, this was followed by a progressive and prominent depletion of the white pulp lymphoid elements, which increased in severity from 21 to 28 dpi, consistent with the observed systemic immunosuppression (Fig. 12).

## Discussion

Chicken infectious anemia virus continues to be a formidable challenge to the poultry industry worldwide, primarily due to its profound immunosuppressive effects, which trigger vaccination failures and heighten susceptibility to co-infections [6, 11, 42]. In Egypt, particularly in the southern (Upper Egypt) governorates, the poultry industry relies heavily on intensive vaccination programs to mitigate the impact of immunosuppressive viruses. Despite the widespread implementation of these protocols, field outbreaks of chicken infectious anemia (CIA) continue to be reported, manifesting as significant economic losses and recurrent vaccination failures. Crucially, while several studies have characterized CIAV in Northern Egypt, there is a profound lack of molecular and pathogenic data concerning the strains circulating in Upper Egypt. The present study was therefore conducted to bridge this knowledge gap, providing the first comprehensive investigation into the prevalence, genetic evolution, and pathogenicity of CIAV in this geographically distinct and high-density poultry sector.

The present study reveals a high prevalence rate of 30% (120/400) via TaqMan-based qPCR across Upper Egyptian governorates. Interestingly, this prevalence is notably higher than the 23% we recently reported for the same period (2024–2025) in other Egyptian provinces [6]. This discrepancy suggests that CIAV may be circulating more intensively in the southern regions (Upper Egypt), potentially due to differences in environmental stressors, flock management practices, or the unique evolutionary dynamics of the strains localized in these governorates. Crucially, while previous research has extensively focused on Northern Egypt, this study represents the first comprehensive investigation into the molecular and pathogenic landscape of CIAV specifically in Upper Egypt. The recorded higher prevalence in this region, despite the concurrent vaccination efforts, underscores a critical "geographical gap" in vaccine efficacy and highlights the urgent need for regional-specific surveillance and the potential development of immunogens that match the highly prevalent genotype II strains identified in our study.

Whole-genome phylogenetic analysis identified four major CIAV genotypes (I–IV), each supported by higher bootstrap values. Specifically, the phylogenetic analysis based on complete genome sequencing demonstrated that five investigated CIAV isolates clustered tightly within genotype II. These isolates shared high nucleotide and amino acid similarities (97–98%) with previous CIAV Egyptian strains isolated in 2015–2016. This observation is consistent with earlier reports mentioned by [3, 15, 43, 44], confirming the continued dominance of genotype II in Egypt. Moreover, there is close genetic relatedness of current Egyptian CIAV isolates and those reported worldwide, including those from China, Australia, Malaysia, the USA, and Brazil, with nucleotide and amino acid similarities ranging from 96 to 98%. These findings are in agreement with previous global studies by [5, 20, 45, 46]. Recently, several commercial vaccines, including Del-Ros, Nobilis P4, and Cux-1, are widely used in breeder flocks to induce maternal antibodies that protect young chicks during their first two to three weeks of life [5, 46]. The phylogenetic analysis and amino acid analyses revealed that these field isolates were distinctly apparent from the vaccines’ strains (CAV 26P4/Netherland-2007), showing 85%–87% amino acid similarity. These previous observations also align with those findings recorded by [15, 47]. This divergence likely contributes to the CIAV infection occurrence in vaccinated flocks; these findings agree practically with studies reported by [3, 15]. Further studies are required to determine the vaccine efficacy against these recent isolates. In contrast, the mentioned isolates exhibited higher similarity (95–98%) to other CIAV vaccine-related strains, including CAV/Del-Ros, CAV Cuxhaven-1, and CAV vaccine 3711.

However, it is critical to interpret this genetic similarity with caution. While a high percentage of nucleotide identity is often observed, it does not provide definitive evidence of direct vaccine derivation or vaccine-derived evolution. Instead, this high similarity reflects a shared evolutionary ancestry or the continuous co-circulation of phylogenetically related ancestral strains within the region, rather than a direct transmission or reversion to virulence of commercial vaccine viruses. Finally, these promising findings confirm continuous circulation of genotype II of CIAV in Egyptian poultry flocks with various mutations and multiple evolutions, particularly within the VP1. Moreover, nucleotide and amino acid sequence analyses demonstrated a high similarity and strong genetic relatedness between these sequences (99%), suggesting the circulation of a single CIAV strain in chicken populations and a common ancestral source. Collectively, these results demonstrate that our current strains circulating in various Egyptian governorates are phylogenetically related to global strains and previously reported Egyptian ones, indicating substantial local circulation alongside broader intercontinental connectivity.

Regarding our available results, the Egyptian CIAV isolate analysis demonstrates an amino acid variation level. The high level of sequence conservation in the N-terminal region aligns with previous studies that suggest this domain is structurally limited and essential for viral integrity [48–50]. Commonly, the substitution at position ~ 16 is detected between global CIAV strains. The presence of leucine substitutions at position 126 in PX596998 may affect capsid shape and antigenic properties. These changes could influence how the host’s immune system recognises the virus and may aid in immune evasion, and these results came nearly in accordance with [51, 52]. The central region of VP1 (aa 220–240), which exhibited variability among the isolates, is under positive selection pressure [53–55]. The isoleucine substitutions and variations within the P/S/L/HG motif may indicate adaptive evolution driven by host immune responses, particularly in vaccinated poultry. The partial similarity of these motifs to those in genotype III strains suggests some convergent evolution. The conserved methionine substitution at position 254 has been previously noted in Egyptian and international strains and is not considered a major virulence factor, parallel to our results [56]. However, the additional leucine substitution in PX596998 may affect protein folding and stability.

Subsequently, the unique cysteine substitution identified in PX596998 is significant, as this region overlaps with VP2-associated functional domains. VP2 plays a crucial role in viral replication and proper VP1 folding. The introduction of a cysteine residue may change protein–protein interaction, potentially impacting the efficiency of viral replication [49, 57]. Concerning previous studies, these mutations occur outside the classic virulence-associated residues of VP1 (positions 75, 89, 125, 139, and 144), indicating that their role is related to structural adaptation. This region, known for its critical virulence-associated residues, was completely conserved compared to the reference strain. The isoleucine substitution in the C-terminal region (~ aa 540) seems to be a lineage-specific marker. Our subsequent results came in accordance with several recent CIAV strains [48, 55]. Additionally, this mutation analysis of these Egyptian isolates supports the ongoing evolution of genotype II CIAV strains circulating in Egypt. Among the examined isolates, PX596998 exhibited the most divergence, containing multiple unique substitutions across different functional regions. This isolate may be an emerging variant with potentially altered biological characteristics.

Genetic recombination significantly influenced virus evolution, playing a key role in generating and maintaining viral genetic diversity [58, 59]. Recombination analysis of the CIAV genotype II isolate PS96998 (Matrouh_Sample5) revealed high nucleotide similarity to both parental strains: CIAV strain 12-GD2018101 and CAV-EG-14. This high sequence identity suggests recombination occurred between closely related genotype II lineages, consistent with recent reports highlighting frequent intra-genotypic recombination in CIAV. Two recombination breakpoints were detected, reflecting the circular structure of the CIAV genome. Importantly, this configuration implies a mosaic genomic structure where the central region originates from one parental lineage, whereas the other regions are from the alternate lineage. This observation is also stated in CIAV recombination by [49–52, 56]. Furthermore, the close relationship between the parental strains and geographically linked isolates may indicate the co-circulation of multiple CIAV lineages in poultry. Such conditions facilitate the emergence of novel variants and promote their recombination. Similar mechanisms have been mentioned in recent CIAV studies from Asia and South America [49, 51, 59].

Regarding homology modelling, the structural features observed in this study align with the role of VP1 as the major capsid protein responsible for virion assembly and antigenicity. Notably, VP1 is the only structural protein forming the capsid of CIAV and is essential for inducing neutralising antibodies [60, 61]. The compact structure observed in this model supports its function in forming a stable icosahedral capsid; these results were consistent with previous structural and functional analyses [61]. The combination of structured (α-helical) and unstructured (loop) regions reflects a typical viral capsid design. The rigid domains provide structural integrity, and flexible loops mediate host interactions and immune evasion. This is particularly important for CIAV, where minor amino acid variations in VP1 significantly influence virulence and antigenicity [60, 62, 63]. Although genomic and phylogenetic analyses demonstrated a close evolutionary relationship between several Egyptian isolates and vaccine-derived strains, functional antigenic characterization was not performed. Future investigations, employing virus-neutralization assay, are warranted to evaluate the extent of immunological cross-reactivity and protective efficacy of currently used genotype I vaccines against the circulating genotype II Egyptian CIAV isolates.

The in vivo pathogenicity trial further underscored the "hypervirulent" nature of the Assiut Genotype II isolate. The manifestation of severe clinical symptoms and the pathognomonic pale, fatty bone marrow were quantitatively corroborated by a drastic decline in packed cell volume (PCV%), which fell to 18 ± 0.58%. This correlation underscores the profound haematological impact of the viral infection. This reduction is notably more pronounced than the 19.33% decline documented in our previous studies [6, 23, 64] and falls well below the established clinical anaemia threshold [65]. Such acute haematopoietic failure is directly linked to the exceptionally high viral titres identified in the bone marrow (9.00 ± 0.22 log₁₀) and thymus (9.50 ± 0.25 log₁₀), where CIAV aggressively targets erythroid and myeloid progenitor cells [64].

Furthermore, the experimental infection resulted in a "total humoral collapse", evidenced by the rapid decline of ELISA antibody titres to undetectable levels by the 4th and 5th weeks post-infection. This immunological exhaustion, which aligns with the patterns observed in our previous study [2, 6, 42], suggests that these emerging Egyptian genotype II variants create a persistent "immunological void". Such a state not only hinders the host’s ability to clear CIAV but also leaves the birds highly vulnerable to secondary opportunistic pathogens, explaining the high mortality and production losses observed in the dense poultry sectors of Upper Egypt.

The dynamics of viral shedding are critical for the horizontal transmission and endemic persistence of CIAV in poultry environments. In the present study, the high viral loads quantified by qPCR across multiple organs, peaking at 9.50 ± 0.25 log₁₀ in the thymus and 9.00 ± 0.22 log₁₀ in the bone marrow, suggest a massive systemic replication that facilitates prolonged viral shedding.

Consistent with our previous observations in [6], the high viral concentration in the lymphoid organs and digestive tract (evidenced by proventricular lesions) indicates that these genotype II strains are shed in high titres through faeces and feather dander. This extensive shedding, coupled with the virus’s well-known environmental stability, explains the rapid spread within flocks and the difficulty of decontamination in Upper Egyptian farms. Furthermore, the persistent high viral load detected in the bone marrow up to 21 dpi suggests that infected birds remain active "shedders" for an extended period, creating a continuous source of infection that can bypass maternally derived antibodies (MDA) in subsequent cycles. This "prolonged shedding profile" is a key factor in the economic impact of genotype II, as it ensures the maintenance of a high viral pressure in the environment, even in the presence of vaccination.

The macroscopic lesions observed in this study provide a clear visual confirmation of the high virulence of the Upper Egyptian CIAV genotype II isolate. The appearance of "blue-wing disease" (Fig. 2A) and extensive systemic haemorrhages in the skeletal muscles and proventriculus (Fig. 2B, C) are indicative of severe vascular damage and thrombocytopenia. These findings align with our recent report [6], suggesting that genotype II strains circulating in Egypt have a high tropism for vascular endothelial cells and haematopoietic tissues, leading to the clinical manifestation of the haemorrhagic syndrome. A pivotal diagnostic feature was the transformation of the bone marrow from its normal deep red colour to a pale, fatty, and yellowish appearance (Fig. 2D). This gross change is a direct consequence of bone marrow aplasia, where the virus destroys the haemocytoblasts. This finding is quantitatively supported by the profound drop in PCV% (18 ± 0.58%) recorded in our results. Consistent with [6, 23], the severity of bone marrow lesions in genotype II infections appears to be more aggressive than those typically associated with the classical genotype I strain, explaining the higher mortality and morbidity rates observed in the field.

The irreversible damage caused by emerging Egyptian genotype II variants is evidenced by systemic lymphoid exhaustion and the hallmark presence of intranuclear inclusion bodies [6, 66, 67]. Furthermore, the direct correlation between maximum viral titres and the development of severe tissue aplasia highlights the enhanced virulence and rapid replication dynamics that characterise these native strains. To mitigate the devastating effects of CIAV in Egypt, developing genotype-matched vaccines based on local isolates is crucial. This approach addresses the limitations of current genotype I-based programmes, particularly the risk of vaccine escape and suboptimal cross-protection. Additionally, the widespread use of Genotype I vaccines may be driving selective pressure that favours the dominance of Genotype II strains, which possess superior environmental stability and fitness in the local field. Such evolutionary shifts necessitate an immediate update to regional vaccination strategies.

Collectively, the scarcity of molecular, and epidemiological data from Upper Egypt has limited the characterization of locally circulating CIAV strains. The present study demonstrates that several Upper Egyptian isolates exhibit notable genetic divergence from vaccine-related strains, particularly within genomic regions associated with viral evolution. While the current data do not directly demonstrate reduced vaccine efficacy, these findings suggest a hypothesis that ongoing viral evolution may influence antigenic similarity between field and vaccine strains. Consequently, continuous molecular surveillance in Upper Egypt is essential not only for recording viral diversity but also for identifying emerging variants that warrant further evaluation through cross-neutralization and vaccine-challenge studies to determine their potential impact on vaccine-mediated protection.

## Conclusion

This study provides a comprehensive pathogenic and genetic characterization of CIAV genotype II strains circulating in commercial broiler flocks across various Egyptian governorates. Genetic detection through PCR and quantitative PCR confirmed the high viral prevalence, while complete genome sequencing of representative isolates revealed 97%–98% and 96%–98% nucleotide and amino acid similarities to reference Egyptian strains (2015–2016) and contemporary global isolates from different countries, respectively. Conversely, a lower genetic similarity (85%–87%) was observed with the commercial vaccine strain ‘CAV 26P4/Netherland-2007’. A major strength of this study is its integrated approach, combining clinical pathogenicity, molecular characterization, and phylogenetic analysis to provide novel genetic insights into the viral epidemiology of Upper Egypt, which has remained poorly investigated. While whole-genome characterization was focused on a selected representative subset of high-viral-load strains, which may limit absolute population-wide genetic representativity, the high sequence identity underscores a synchronized local evolution. These promising findings provide empirical evidence of the continuous circulation, active mutations, and ongoing evolution of genotype II variants in Egyptian poultry. Future investigations should focus on experimental vaccine-matching trials to update national prevention and enhance regional control strategies.

## Supplementary Information


Additional file 1.


## Data Availability

All data are available upon request.

## References

[1] Schat KA, van Santen VL. *Chicken infectious anemia* and circovirus infections in commercial flocks. In: Swayne DE, editor. Diseases of poultry. 14th ed. Hoboken, NJ: Wiley-Blackwell; 2020. p. 284–320.

[2] Alispahic A, Softic A, Kustura A, Omeragic J, Goletic T Clinical and welfare aspects of immunosuppression in poultry farming From Zoo to Farm-The Quest for Animal Welfare, IntechOpen, London, UK 2024; pp. 1–20.

[3] Abdel-Mawgod S, Adel A, Arafa AS, Hussein HA. Full genome sequences of chicken anemia virus demonstrate mutations associated with pathogenicity in two different field isolates in Egypt. Virusdisease. 2018;29(3):333–41. 10.1007/s13337-018-0467-z. 30159368 PMC6111964

[4] Adair BM. Immunopathogenesis of chicken anemia virus infection. Dev Comp Immunol. 2000;24(2–3):247–55. 10717291 10.1016/s0145-305x(99)00076-2

[5] Song H, Kim H, Kwon Y, Kim H. Genetic characterization of chicken infectious anaemia viruses isolated in Korea and their pathogenicity in chicks. Front Cell Infect Microbiol. 2024;14:1333596. 10.3389/fcimb.2024.1333596. 38420609 PMC10900523

[6] Soliman YA, MS El-Nagar Eman, AN Gamal Maha MS, Khattab M, Salem Heba Mohamed G, Seadawy and Doha Abd Alrahman Ahmed. "Molecular Characterization, Evolutionary Dynamics, and Pathogenic Assessment of Chicken Infectious Anemia Virus Isolates Circulating in Egyptian Poultry." *Poultry Science* 2026; 106906. 10.1016/j.psj.2026.106906.PMC13141756 42061267

[7] McNulty MS, Connor TJ, McNeilly F, McLoughlin MF, Kirk- patrick KS. Preliminary characterization of isolates of chicken anaemia agent from the United Kingdom. Avian Pathol. 1990;19:67–73. 10.1080/03079459008418657 18679915

[8] Spackman E, Cloud SS, Rosenberger JK. Comparison of a putative second serotype of chicken infectious anemia virus with a prototypical isolate II. Antigenic and physicochemical charac- teristics. Avian Dis. 2002;46:956–63. 12495057 10.1637/0005-2086(2002)046[0956:COAPSS]2.0.CO;2

[9] Yan T, Song Y, Zhang D, Wang Z, Li R, Zhang X, et al. Chicken anemia virus inhibits hematopoiesis and development of chicken embryo. Poult Sci. 2025;104(4):104857. 10.1016/j.psj.2025.104857. 40036936 PMC11926702

[10] -Yuasa N. Propagation and infectivity titration of the Gifu-1 strain of chicken anemia agent in a cell line (MDCC-MSB1) derived from Marek’s disease lymphoma. Natl Inst Anim Health Q (Tokyo). 1983; Spring; 23(1):13–20. 6680769

[11] Karki M, Bora M, M M, Kumar A. An updated review on *chicken infectious anaemia*. World Poult Sci J. 2025;81:117–34.

[12] Hussein HA, Sabry MZ, El-Sanousi AA, El-Sabagh IM. Chicken anemia virus in broiler chickens in Egypt: molecular and pathological studies. Journal of the Egyptian Veterinary Medical Association. 2003;63:95–106.

[13] Elsamadony HA, Mekky HM, Fedawy HS, Sedeek DM, El-Shemy A, Saad AS. Genetic differences and pathogenicity of chicken anemia virus strains in broiler’s baby chicks. Egypt J Vet Sci. 2025;56(4):851–61. 10.21608/EJVS.2024.256791.1736.

[14] Wu X, Kong J, Yao Z, Sun H, Liu Y, Wu Z, et al. A new rapid and sensitive method for detecting chicken infectious anemia virus. Front Microbiol. 2022;13:994651. 36246275 10.3389/fmicb.2022.994651 PMC9558101

[15] Erfan AM, Selim AA, Naguib MM. Characterization of full genome sequences of chicken anemia viruses circulating in Egypt reveals distinct genetic diversity and evidence of recombination. Virus Res. 2018;251:78–85. 10.1016/j.virusres.2018.05.008. 29751020

[16] Hall TA. BioEdit: a user-friendly biological sequence alignment editor and anarogram for Windows 95/98/NT. Nucl Acids Symp Ser. 1999;41:95–8.

[17] Tamura K, Peterson D, Peterson N, Stecher G, Nei M, Kumar S. Molecular evolutionary genetics analysis using maximum likelihood, evolutionary distance, and maximum parsimony methods. Mol Biol Evol. 2011;28:2731–9. 10.1093/molbev/msr121. 21546353 PMC3203626

[18] Lole KS, Bollinger RC, Paranjape RS, Gadkari D, Kulkarni SS, Novak NG, et al. Full-length human immunodeficiency virus type 1 genomes from subtype C-infected seroconverters in India, with evidence of intersubtype recombination. J Virol. 1999;73:152–60. 10.1128/JVI.73.1.152-160.1999. 9847317 PMC103818

[19] Martin DP, Murrell B, Golden M, Khoosal A, Muhire B. RDP4: Detection and analysis of recombination patterns in virus genomes. Virus Evol. 2015; 1:vev003. 10.1093/ve/vev003 PMC5014473 27774277

[20] Xu S, Zhang Z, Xu X, Ji J, Yao L, Kan Y, Xie Q, Bi Y Molecular Characteristics of Chicken Infectious Anemia Virus in Central and Eastern China from 2020 to 2022. Animals 2023; 13, 2709. https:// doi.org/10.3390/ani13172709 PMC10487239 37684973

[21] Zhang Q, Shi H, Liu G, Yao Z, Liu H, Wei L, et al. Serological investigation of infectious anemia in Wenchang chickens in Hainan province. Prog Vet Med. 2024;45:121–5.

[22] Lu Y, Li W, Liu Y, Lin J, Luo H, Wang Y, et al. Epidemiological characteristics and genetic diversity of chicken infectious anemia virus (CIAV) in Guangdong Province, China. Vet Sci. 2025;12:972. 10.3390/vetsci12100972. 41150112 PMC12567861

[23] AbdAlla HA, Tantawy LAEG, Hussein HA, Elsamadony HA, Hassan NRA. Pathogenesis of two chicken infectious anaemia virus (CIAV) vaccines in lymphoid and non-lymphoid organs. Vet Med J (Giza). 2022;68:1–24.

[24] Schat KA. Chicken anemia virus. Curr Top Microbiol Immunol. 2009;331:151–83. 10.1007/978-3-540-70972-5_10. 19230563

[25] Shao H, Li J, Zhang J, Zhang Q, Ma L, Lu J, et al. Research note: A novel peptide-based ELISA for efficient detection of antibody against chicken infectious anemia virus. Poult Sci. 2023;102:102284. 36399931 10.1016/j.psj.2022.102284 PMC9673107

[26] Su Z, Leng M, Wu Z, Chen W, Yang S, Li X, et al. From surveillance to pathogenesis: characterization of genotype V of chicken infectious anemia virus. Front Vet Sci. 2025;12:1710392. 41340930 10.3389/fvets.2025.1710392 PMC12670581

[27] Mirzaiee K, Shoushtari A, Bokaie S, Fallah Mehrabadi MH, Peighambari SM. Trend of Changes in the Titer of Antibody against Avian Influenza Virus H9n2 during Raising Period in Vaccinated and Unvaccinated Broiler Farms in Qazvin Province, Iran: A Cohort Study. Arch Razi Inst. 2020;75:9–16. 10.22092/ari.2018.120089.1183. 32291997 PMC8410165

[28] Abdelwhab e-SM, Erfan AM, Grund C, Ziller M, Arafa AS, Beer M, et al. Simultaneous detection and differentiation by multiplex real time RT-PCR of highly pathogenic avian influenza subtype H5N1 classic (clade 2.2.1 proper) and escape mutant (clade 2.2.1 variant) lineages in Egypt. Virol J. 2010;7:260. 10.1186/1743-422X-7-260. 20929539 PMC2958913

[29] Shosha EAE, Mohamd MK, Shehata MAE, Mohamed MH, Eldaghayes I, and Abdelhafez MS. Genomic and evolutionary characterization of newly emerged highly pathogenic avian influenza H5N1 clade 2.3.4.4b viruses circulating in broiler flocks in Upper Egypt (2023–2025). *Veterinary World*, 18(12), 3745–3760 2025; 10.14202/vetworld.2025.3745-3760. PMC12913964 41716167

[30] Wise MG, Suarez DL, Seal BS, Pedersen JC, Senne DA, King DJ, et al. Development of a real-time reverse-transcription PCR for detection of newcastle disease virus RNA in clinical samples. J Clin Microbiol. 2004;42:329–38. 10.1128/JCM.42.1.329-338.2004. 14715773 PMC321685

[31] Mosad SM, Elsayed MM, Hammad EM, Hendam BM, Ali HS, Eladl AH, et al. Genotype classification and pathogenicity of infectious bursal disease virus circulating in vaccinated broiler chicken farms. Vet Res Commun. 2024;48:3089–104. 10.1007/s11259-024-10468-z. 39078474 PMC11442545

[32] Cheng MC, Lai GH, Tsai YL, Lien YY. Circulating hypervirulent Marek’s disease viruses in vaccinated chicken flocks in Taiwan by genetic analysis of *meq* oncogene. PLoS ONE. 2024;19:e0303371. 10.1371/journal.pone.0303371. 38728352 PMC11086920

[33] Shosha EAE, Eldaghayes I, Abdel-Rahaman SEA, Hussein A, El Naggar HM, Gamaleldin MA, et al. Pathogenicity and genotyping of fowl adenovirus-D serotype 2/11 circulating in commercial broilers in Egypt. Viruses. 2026;18(2):252. 10.3390/v18020252. 41754595 PMC12944925

[34] Shosha EAM, Zanaty AM, Abdallah AF, Eldaghayes I. Histopathological diagnosis and genetic diversity of avian encephalomyelitis virus circulating in broiler farms in Egypt. Open Vet J. 2026;16(2):1338–50. 10.5455/OVJ.2026.v16.i2.50. 42376377 PMC13313924

[35] Sallam M, Mysara M, Benotmane M.A, Tamarat R, Santos SCR, Crijns APG, Spoor D, Van Nieuwerburgh F, Deforce D, Baatout S, et al. Correction: Sallam et al. DNA Methylation Alterations in Fractionally Irradiated Rats and Breast Cancer Patients Receiving Radiotherapy. *Int. J. Molecular Sciences* 2023; 24, 17590. 10.3390/ijms242417590 PMC9783664 36555856

[36] Nahar A, Awasthi SP, Hatanaka N, Okuno K, Hoang PH, Hassan J, et al. Prevalence and characteristics of extended-spectrum β-lactamase-producing *Escherichia coli* in domestic and imported chicken meats in Japan. J Vet Med Sci. 2018;80:510–7. 10.1292/jvms.17-0708. 29434117 PMC5880835

[37] Palmeira A, Santos LR, Borsoi A, Rodrigues LB, Calasans M, Nascimento VP. Serovars and antimicrobial resistance of *Salmonella* spp. isolated from turkey and broiler carcasses in Southern Brazil between 2004 and 2006. Rev Inst Med Trop Sao Paulo. 2004;58:19. 10.1590/S1678-9946201658019. PMC4804556 27007562

[38] Ahmed DAA, Hassan AK, Ali DAA, Shosha EAE, Eldaghayes IM, Khair SMAA. Molecular detection and sequencing of the NetB toxin gene of *Clostridium perfringens* and evaluation of its pathogenicity in broiler chicken. Poult Sci. 2026;105:106289. 10.1016/j.psj.2025.106289. 41456403 PMC12803810

[39] Rufai AA, Zakaria Z, Yu CY, Ganapathy K, Abu J, Ahmad NI, et al. Prevalence and antimicrobial susceptibility of *Mycoplasma gallisepticum* and *Mycoplasma synoviae* isolated from the central peninsular Malaysia. Poult Sci. 2025;104:105552. 10.1016/j.psj.2025.105552. 40749628 PMC12336823

[40] Brakstad OG, Aasbakk K, Maeland JA. Detection of *Staphylococcus aureus* by polymerase chain reaction amplification of the *nuc* gene. J Clin Microbiol. 1992;30:1654–60. 10.1128/jcm.30.7.1654-1660.1992. 1629319 PMC265359

[41] Wódz K, Chodkowska KA, Iwiński H, Różański H, Wojciechowski J. In vitro evaluation of phytobiotic mixture antibacterial potential against *Enterococcus* spp. strains isolated from broiler chicken. Int J Mol Sci. 2024;25:4797. 10.3390/ijms25094797. 38732016 PMC11084370

[42] Pohuang T, Worawong K, Sarachu K, Khunbutsri D, Junnu S. Molecular characterization and phylogenetic diversity of fowl aviadenovirus serotype 8b associated with inclusion body hepatitis in Thai chickens. Vet World. 2025;18:1685. 40689183 10.14202/vetworld.2025.1685-1693 PMC12269957

[43] Abdelhalim A, Samir A, Yehia N. Molecular characterization of chicken anaemia virus circulating in commercial poultry flocks in Egypt during 2020. World Vet J. 2021;11(2):235–41.

[44] Abdel-Mawgod S, Zanaty A, Elhusseiny M, Said D, Samir A, Elsayed MM, et al. Genetic heterogeneity of chicken anemia virus isolated in selected Egyptian provinces as a preliminary investigation. Front Vet Sci. 2024;11:1362219. 10.3389/fvets.2024.1362219. 38840626 PMC11150715

[45] Di Francesco A, Quaglia G, Salvatore D, Sakhria S, Catelli E, Bessoussa G, et al. Occurrence of chicken infectious anemia virus in industrial and backyard Tunisian broilers: preliminary results. Animals. 2022;12:62. 10.3390/ani12010062. PMC8749614 35011168

[46] Prezotto C, Marin S, Araújo T, Barbosa F, Barrios P, Gomes A, et al. Experimental coinfection of chicken anemia virus and *mycoplasma gallisepticum* vaccine strains in broiler chicks. Rev Bras Cienc Avic. 2016;18:475–80. 10.1590/1806-9061-2016-0235.

[47] Aboelkhair M, El-Razak A, and Metwally A Molecular characterization of chicken anemia virus circulating in chicken flocks in Egypt 2014; Advances in Virology, Article ID 797151. 10.1155/2014/797151 PMC4180199 25302064

[48] Ou SC, Lin YJ, Wang YC, Chen HJ, Shien JH. Molecular evolution and antigenic variation of chicken anemia virus VP1 gene in Asia. Arch Virol. 2020;165:1543–53.

[49] Zhang X, Liu Y, Wu B, Sun B, Chen F. Genetic diversity and molecular evolution of chicken anemia virus in China from 2014 to 2019. Viruses. 2021;13:1123.34207958

[50] Li Y, Fang L, Cui S, Fu J, Li X. Molecular characterization and phylogenetic analysis of chicken anemia virus isolates in recent outbreaks. Front Vet Sci. 2022;9:876543.

[51] Dos Santos HF, Knak MB, da Silva AD, Canal CW. Whole-genome sequencing reveals genetic variability and recombination in chicken anemia virus. Infect Genet Evol. 2020;81:104268.32126303

[52] Nguyen VG, Kim HK, Moon HJ, Jang YS, Lee HS. Evolutionary dynamics of chicken anemia virus in Southeast Asia. Transbound Emerg Dis. 2021;68:2663–74.

[53] Elbestawy AR, Abd El-Hamid HS, Ellakany HF, Gado AR, El-Rayes SH, Salaheldin AH. Molecular characterization and genetic diversity of chicken anemia virus circulating in Egypt. *Animals* 2020; 10, 2307.

[54] Shah PT, Bahoussi AN, Cui X, Shabir S, Wu C, Xing L. Genetic diversity, distribution, and evolution of chicken anemia virus: a comparative genomic and phylogenetic analysis. Front Microbiol. 2023;14:1145225. 10.3389/fmicb.2023.1145225. 36970671 PMC10034120

[55] Huang Z, Wei K, Zhang Y, Wang J, Liu B, Shao H, et al. Genomic characterization of chicken anemia virus in broilers in Shandong Province, China, 2020–2021. Front Vet Sci. 2022;9:816860. 10.3389/fvets.2022.816860. 35372548 PMC8968957

[56] Wang D, Fan W, Han G, et al. Insights into capsid protein evolution and host adaptation of chicken anemia virus. J Gen Virol. 2023;104:001789.

[57] Noteborn MHM, Verschueren CAJ, Koch G, Van der Eb AJ. Simultaneous expression of recombinant baculovirus-derived chicken anemia virus (CAV) proteins VP1 and VP2 is required for formation of the CAV-specific neutralizing epitope. J Virol. 1998;72(1):619–23. 10.1099/0022-1317-79-12-3073 9880024

[58] Tan C, Wang Z, Lei X, Lu J, Yan Z, Qin J, et al. Epidemiology, molecular characterization, and recombination analysis of chicken anemia virus in Guangdong province, China. Arch Virol. 2020;165:1409–17. 10.1007/s00705-020-04604-8. 32318833

[59] Liu L, Li Y, Yin M, Zhao P, Guo L, Wang Y. Genomic characterization of chicken anemia virus in broilers in Shandong Province, China, 2020–2021. Front Vet Sci. 2022;9:816860. 10.3389/fvets.2022.816860. 35372548 PMC8968957

[60] Prasetyo AA, et al. Structural insights into VP1 protein variability and its role in CIAV pathogenicity. Front Microbiol. 2021;12:681838. 10.3389/fmicb.2021.681838.

[61] Ou S-C, Lin Y-J, Lin C-C, Liu P-C, Wang C-Y. Structural and Functional Characterization of the Capsid Protein VP1 of Chicken Infectious Anemia Virus. Viruses. 2022;14:2115. 10.3390/v14102115. 36298670 PMC9612391

[62] Zhou J, Engelhardt OG, Young JAT, et al. Functional analysis of VP1 protein of chicken infectious anemia virus. J Gen Virol. 2001;82:219–29. 10.1099/0022-1317-82-1-219.

[63] Wang D, Fan W, Han G, Li H, Wang J, Wang Y. Molecular characterization and phylogenetic analysis of chicken infectious anemia virus in China. Infect Genet Evol. 2022;97:105156. 10.1016/j.meegid.2021.105156.

[64] Mostafa DIA, Hamed RI, Salem SM, Abdallah F, Tolba HMN. Pathological and immunopathological studies on broiler chicks infected with *Chicken anemia virus*. Adv Anim Vet Sci. 2021;9:508–18.

[65] Govindhasamy, S. Studies on pathology of lymphoid organs in chickens with special emphasis on immunosuppressive viral diseases 2022; PhD Diss. Indian Veterinary Research Institute, Bareilly, India.

[66] Rimondi A, Pinto S, Olivera V, Dibárbora M, Pérez-Filgueira M, Craig MI, et al. Comparative histopathological and immunological study of two field strains of chicken anemia virus. Vet Res. 2014;45:102. 10.1186/s13567-014-0102-y. 25294222 PMC4207886

[67] Nighil Rosh AS. Apoptosis Induced by Recombinant Plasmids Encoding *Chicken Anemia Virus VP3* and Canine Parvovirus *NS1* Genes in Bovine Tumor Cells. Indian Veterinary Research Institute, Bareilly, India (2015); PhD Diss.

