# Correction: Full genome sequencing, evolutionary dynamics, and pathogenicity evaluation of chicken infectious anemia virus with emphasis on Upper Egypt reveals genetic variability linked to vaccinal strains

**DOI:** 10.1186/s12985-026-03291-2

**Published:** 2026-08-29

**Authors:** Eman Abd Elmenum Shosha, Ibrahim Eldaghayes, Ali Mahmoud Zanaty, Mohammed A. Gamaleldin, Mervat Masoud Mohamed, A. N. Gamal Maha, Doha Abd Alrahman Ahmed

**Affiliations:** 1 Virology Department, Faculty of Veterinary Medicine, New Valley University, El- Khargia, 72511 Egypt; 2 Department of Microbiology and Parasitology, Faculty of Veterinary Medicine, University of Tripoli, P.O. Box 13662, Tripoli, Libya; 3 Reference Laboratory for Quality Control On Poultry, Agriculture Research Center (ARC), Animal Health Institute (AHRI), Giza, 12619 Egypt; 4 Department of Poultry Disease, Animal Health Research Institute, Agricultural Research Center, Assiut, 71526 Egypt; 5 Central Laboratory for Evaluation of Veterinary Biologics (CLEVB), Agricultural Research Center (ARC), Cairo, 11381 Egypt; 6 Department of Avian and Rabbit Medicine, Faculty of Veterinary Medicine, Assiut University, Assiut, Egypt

**Correction: Virology Journal (2026) 23:179**

**https://doi.org/10.1186/s12985-026-03257-4**

In this article [1], the author’s name Mohammed A. Gamaleldin was incorrectly written as Mohammed A. Gamal Eldin.

The original article has been corrected.
