# Supplementary material for: Full genome sequencing, evolutionary dynamics, and pathogenicity evaluation of chicken infectious anemia virus with emphasis on Upper Egypt reveals genetic variability linked to vaccinal strains
Source: Virol J. 2026 Jul 24;23:179. doi: 10.1186/s12985-026-03257-4 (PMC13401295; doi:10.1186/s12985-026-03257-4)
Supplement: Supplementary file 1 — Additional file 1. [file 12985_2026_3257_MOESM1_ESM.docx]

| **Number** | **Isolates** | **Accession number** | **Isolation date (yr)** | **Geographic location/provinces** | **Age/Week** | **Type of Samples** | **Total No. of samples** | **Total No. of +v samples** |
| --- | --- | --- | --- | --- | --- | --- | --- | --- |
| **1** | CAV_Partial_Matrouh7 | PX776521 | 2024 | Matrouh | 22wks | Liver, Spleen | 40 | 10 |
|  | CAV_Fullgenome_Matrouh_Sample5 | PX596998 |  |  |  |  |  |  |
| **2** | CAV_Partial_Cairo6 | PX776520 | 2025 | Cairo | 2wks | Bursa, Thymus | 40 | 15 |
|  | CAV_Fullgenome_Cairo_Sample4 | PX596997 |  |  |  |  |  |  |
| **3** | CAV_Partial_ Assiut_sample1 | PX597000 | 2025 | Assiut | 27wks | Liver, Spleen | 60 | 20 |
|  | CAV_Fullgenome_ Assiut_Sample1 | PX596994 |  |  |  |  |  |  |
| **4** | CAV_Partial_NewValley3 | PX776517 | 2024 | New Valley | 3wks | Bursa, Thymus | 35 | 10 |
|  | CAV_Fullgenome_NewValley_Sample3 | PX596996 |  |  |  |  |  |  |
| **5** | CAV_Partial_Qena4 | PX776518 | 2025 | Qena | 2wk | Bursa, Thymus | 35 | 9 |
|  | CAV_Fullgenome_Qena_Sample2 | PX596995 |  |  |  |  |  |  |
| **6** | CAV_Partial_Sohag2 | PX776516 | 2025 | Sohag | 2wk | Bursa, Thymus | 35 | 10 |
| **7** | CAV_Partial_Aswan5 | PX776519 | 2024 | Aswan | 4wks | Bursa, Thymus | 35 | 11 |
| **8** | CAV_Partial_Minia8 | PX776522 | 2025 | Minia | 2wk | Bursa, Thymus | 30 | 10 |
| **9** | CAV_Partial_Fayoum9 | PX776523 | 2024 | Fayoum | 22wks | Liver, Spleen | 45 | 10 |
| **10** | CAV_Partial_Kafr_El_Sheikh_Sample10 | PX597002 | 2025 | Kafr_El_Sheikh | 22wks | Liver, Spleen | 45 | 15 |
| **Total** | | | | | | | 400 | 120 (30%) |

**Table S1:** Epidemiological data and GenBank accession numbers of CIAV isolates identified in various Egyptian governorates during 2024–2025.

| ***6*** | *MG827098* | *MG827099* | *DQ124936* | *U65414* | *AB027470* | *DQ217400* | *AF311900* | *MG846491* | *KU645517* | *OL448832* | *AB031296* | *AF311892* | *AF390038* | *MG827100* | *MH001555* | *MT268631* | | *MT268633* | *D10068* | | *AF313470* | | *M81223* | | | *NC_001427* | | *EF683159* | | | | ***PX596994*** | | ***PX596995*** | | | ***PX596996*** | | ***PX596997*** | | | ***PX596998*** | | | |  |  |
| --- | --- | --- | --- | --- | --- | --- | --- | --- | --- | --- | --- | --- | --- | --- | --- | --- | --- | --- | --- | --- | --- | --- | --- | --- | --- | --- | --- | --- | --- | --- | --- | --- | --- | --- | --- | --- | --- | --- | --- | --- | --- | --- | --- | --- | --- | --- | --- |
|  |  |  |  |  |  |  |  |  |  |  | *Nucleotides Identity* |  |  |  |  |  |  | | |  | |  | | |  | |  | |  | |  | | |  | | | |  | | | | | |  | | |  |
| *MG827098: II CAV/2015/Egypt* | *ID* | *98%* | *99%* | *99%* | *99%* | *99%* | *99%* | *99%* | *98%* | *97%* | *97%* | *97%* | *97%* | *97%* | *97%* | *97%* | | *97%* | *86%* | | *97%* | | | *97%* | | | *97%* | | | *96%* | | | *98%* | | *98%* | *98%* | | | | | *98%* | | *97%* | | |  |  |
| *MG827099: II CAV/GZ2-2016/Egypt* | *99%* | *ID* | *98%* | *98%* | *98%* | *97%* | *98%* | *98%* | *98%* | *97%* | *98%* | *98%* | *98%* | *98%* | *98%* | *98%* | | *98%* | *86%* | | *98%* | | | *97%* | | | *97%* | | | *96%* | | | *98%* | | *98%* | *98%* | | | | | *98%* | | *98%* | | |  |  |
| *DQ124936: II CAV/AH4/China* | *99%* | *98%* | *ID* | *99%* | *99%* | *99%* | *99%* | *99%* | *99%* | *97%* | *97%* | *97%* | *97%* | *97%* | *96%* | *96%* | | *96%* | *85%* | | *97%* | | | *96%* | | | *97%* | | | *96%* | | | *98%* | | *98%* | *98%* | | | | | *96%* | | *97%* | | |  |  |
| *U65414: II CAV/704/Australia* | *99%* | *98%* | *99%* | *ID* | *99%* | *99%* | *99%* | *99%* | *99%* | *97%* | *97%* | *97%* | *96%* | *97%* | *96%* | *96%* | | *96%* | *85%* | | *96%* | | | *96%* | | | *96%* | | | *96%* | | | *98%* | | *98%* | *98%* | | | | | *98%* | | *97%* | | |  |  |
| *AB027470: II CAV/TR20/Japan* | *99%* | *98%* | *99%* | *99%* | *ID* | *99%* | *99%* | *99%* | *98%* | *96%* | *97%* | *97%* | *96%* | *97%* | *96%* | *96%* | | *96%* | *85%* | | *96%* | | | *96%* | | | *96%* | | | *96%* | | | *98%* | | *98%* | *98%* | | | | | *98%* | | *97%* | | |  |  |
| *DQ217400: IICAV/SMSC-1P9WT/Malaysia* | *99%* | *98%* | *99%* | *99%* | *99%* | *ID* | *99%* | *99%* | *98%* | *96%* | *96%* | *97%* | *96%* | *96%* | *96%* | *96%* | | *96%* | *85%* | | *96%* | | | *96%* | | | *96%* | | | *95%* | | | *97%* | | *97%* | *97%* | | | | | *97%* | | *97%* | | |  |  |
| *AF311900: II CAV/98D06073/USA* | *99%* | *99%* | *99%* | *99%* | *99%* | *98%* | *ID* | *99%* | *98%* | *97%* | *97%* | *97%* | *96%* | *97%* | *96%* | *96%* | | *96%* | *85%* | | *96%* | | | *96%* | | | *96%* | | | *96%* | | | *98%* | | *98%* | *98%* | | | | | *98%* | | *97%* | | |  |  |
| *MG846491: II CAV/RS/Brazil/15/1R* | *99%* | *99%* | *99%* | *99%* | *99%* | *99%* | *99%* | *ID* | *98%* | *97%* | *97%* | *97%* | *96%* | *97%* | *96%* | *96%* | | *96%* | *85%* | | *97%* | | | *96%* | | | *96%* | | | *96%* | | | *97%* | | *97%* | *97%* | | | | | *97%* | | *96%* | | |  |  |
| *KU645517: II CAV/SD1513/China* | *99%* | *98%* | *99%* | *99%* | *99%* | *99%* | *99%* | *99%* | *ID* | *96%* | *96%* | *96%* | *96%* | *96%* | *96%* | *96%* | | *96%* | *85%* | | *96%* | | | *96%* | | | *96%* | | | *96%* | | | *98%* | | *98%* | *97%* | | | | | *97%* | | *96%* | | |  |  |
| *OL448832: III CAV/SD2001/China* | *98%* | *99%* | *98%* | *98%* | *98%* | *97%* | *98%* | *98%* | *97%* | *ID* | *98%* | *98%* | *98%* | *98%* | *98%* | *98%* | | *98%* | *87%* | | *98%* | | | *98%* | | | *98%* | | | *95%* | | | *97%* | | *97%* | *97%* | | | | | *97%* | | *97%* | | |  |  |
| *AB031296: III CAV/Japan* | *97%* | *98%* | *97%* | *97%* | *97%* | *96%* | *97%* | *97%* | *97%* | *98%* | *ID* | *99%* | *99%* | *99%* | *99%* | *99%* | | *99%* | *87%* | | *99%* | | | *98%* | | | *99%* | | | *96%* | | | *97%* | | *97%* | *97%* | | | | | *95%* | | *95%* | | |  |  |
| *AF311892: III CAV/98D02152/USA* | *97%* | *98%* | *97%* | *97%* | *97%* | *97%* | *97%* | *97%* | *97%* | *99%* | *99%* | *ID* | *99%* | *99%* | *99%* | *99%* | | *99%* | *88%* | | *99%* | | | *99%* | | | *99%* | | | *96%* | | | *97%* | | *97%* | *96%* | | | | | *96%* | | *96%* | | |  |  |
| *AF390038: III CAV/3-1/Malaysia* | *97%* | *98%* | *97%* | *97%* | *97%* | *96%* | *97%* | *97%* | *96%* | *99%* | *98%* | *99%* | *ID* | *99%* | *98%* | *98%* | | *98%* | *87%* | | *98%* | | | *99%* | | | *99%* | | | *96%* | | | *97%* | | *97%* | *97%* | | | | | *96%* | | *96%* | | |  |  |
| *MG827100: III CAV/SK4-2017/Egypt* | *97%* | *98%* | *97%* | *97%* | *97%* | *97%* | *97%* | *98%* | *97%* | *98%* | *99%* | *100%* | *98%* | *ID* | *99%* | *99%* | | *99%* | *87%* | | *100%* | | | *98%* | | | *98%* | | | *96%* | | | *97%* | | *97%* | *97%* | | | | | *95%* | | *95%* | | |  |  |
| *MH001555: III CAV/6/2017EGYPT* | *97%* | *98%* | *97%* | *97%* | *97%* | *96%* | *97%* | *97%* | *97%* | *98%* | *99%* | *99%* | *98%* | *99%* | *ID* | *100%* | | *100%* | *87%* | | *99%* | | | *98%* | | | *98%* | | | *96%* | | | *98%* | | *98%* | *98%* | | | | | *98%* | | *98%* | | |  |  |
| *MT268631: III CAV/Ismailia/2019/Egypt* | *97%* | *98%* | *97%* | *97%* | *97%* | *96%* | *97%* | *97%* | *97%* | *98%* | *99%* | *99%* | *98%* | *99%* | *100%* | *ID* | | *100%* | *87%* | | *99%* | | | *98%* | | | *98%* | | | *96%* | | | *98%* | | *98%* | *98%* | | | | | *96%* | | *96%* | | |  |  |
| *MT268633: III CAV/ElBeheira/2019/Egypt* | *97%* | *98%* | *97%* | *97%* | *97%* | *96%* | *97%* | *97%* | *97%* | *98%* | *99%* | *99%* | *98%* | *99%* | *100%* | *100%* | | *ID* | *87%* | | *99%* | | | *98%* | | | *98%* | | | *96%* | | | *98%* | | *98%* | *98%* | | | | | *98%* | | *96%* | | |  |  |
| *D10068: III CAV 26P4/Netherland-2007* | *86%* | *87%* | *86%* | *86%* | *86%* | *85%* | *86%* | *86%* | *85%* | *87%* | *88%* | *88%* | *87%* | *87%* | *87%* | *87%* | | *87%* | *ID* | | *87%* | | | *87%* | | | *87%* | | | *84%* | | | *86%* | | *86%* | *86%* | | | | | *86%* | | *86%* | | |  |  |
| *AF313470: III CAV/Del Rose/Vaccine* | *97%* | *98%* | *97%* | *97%* | *97%* | *97%* | *97%* | *97%* | *97%* | *98%* | *99%* | *99%* | *98%* | *100%* | *99%* | *99%* | | *99%* | *87%* | | *ID* | | | *98%* | | | *98%* | | | *96%* | | | *97%* | | *97%* | *97%* | | | | | *97%* | | *97%* | | |  |  |
| *M81223: III CAV Cuxhaven1/vaccine* | *97%* | *98%* | *97%* | *97%* | *97%* | *96%* | *97%* | *97%* | *96%* | *99%* | *98%* | *98%* | *99%* | *98%* | *98%* | *98%* | | *98%* | *87%* | | *98%* | | | *ID* | | | *99%* | | | *96%* | | | *97%* | | *97%* | *97%* | | | | | *97%* | | *97%* | | |  |  |
| *NC_001427: III CAV Vaccine* | *97%* | *98%* | *97%* | *97%* | *97%* | *96%* | *97%* | *97%* | *96%* | *99%* | *98%* | *99%* | *99%* | *98%* | *98%* | *98%* | | *98%* | *87%* | | *98%* | | | *99%* | | | *ID* | | | *96%* | | | *97%* | | *97%* | *97%* | | | | | *97%* | | *97%* | | |  |  |
| *EF683159: I CAV vaccine 3711/Australia* | *97%* | *98%* | *97%* | *97%* | *97%* | *97%* | *97%* | *98%* | *97%* | *98%* | *98%* | *99%* | *98%* | *99%* | *98%* | *98%* | | *98%* | *86%* | | *99%* | | | *97%* | | | *97%* | | | *ID* | | | *95%* | | *95%* | *96%* | | | | | *96%* | | *95%* | | |  |  |
| *PX596994: CAV Assiut/1* | *98%* | *98%* | *98%* | *98%* | *98%* | *97%* | *98%* | *98%* | *98%* | *98%* | *98%* | *98%* | *98%* | *98%* | *98%* | *98%* | | *98%* | *85%* | | *98%* | | | *97%* | | | *97%* | | | *97%* | | | *ID* | | *99%* | *99%* | | | | | *99%* | | *99%* | | |  |  |
| *PX596995: CAV Qena/2* | *98%* | *98%* | *98%* | *98%* | *97%* | *97%* | *98%* | *98%* | *97%* | *98%* | *98%* | *98%* | *98%* | *98%* | *98%* | *98%* | | *98%* | *87%* | | *98%* | | | *98%* | | | *98%* | | | *98%* | | | *99%* | | *ID* | *100%* | | | | | *100%* | | *99%* | | |  |  |
| *PX596996: CAV NewValley/3* | *98%* | *98%* | *98%* | *98%* | *97%* | *97%* | *98%* | *98%* | *97%* | *98%* | *98%* | *98%* | *96%* | *98%* | *98%* | *98%* | | *98%* | *87%* | | *98%* | | | *98%* | | | *98%* | | | *98%* | | | *99%* | | *99%* | *ID* | | | | | *100%* | | *99%* | | |  |  |
| *PX596997: CAV Cairo/4* | *98%* | *98%* | *98%* | *98%* | *97%* | *97%* | *98%* | *96%* | *97%* | *98%* | *98%* | *98%* | *98%* | *98%* | *98%* | *98%* | | *98%* | *87%* | | *98%* | | | *98%* | | | *98%* | | | *98%* | | | *99%* | | *99%* | *100%* | | | | | *ID* | | *99%* | | |  |  |
| *PX596998: CAV Matrouh/5* | *97%* | *98%* | *96%* | *97%* | *96%* | *96%* | *96%* | *96%* | *96%* | *98%* | *98%* | *96%* | *98%* | *98%* | *98%* | *98%* | | *98%* | *85%* | | *98%* | | | *97%* | | | *97%* | | | *97%* | | | *98%* | | *98%* | *99%* | | | | | *99%* | | *ID* | | |  |  |
|  |  |  |  |  |  |  |  |  |  |  |  |  |  | *Amino acid identity* |  |  |  | | |  | |  | | |  | |  | |  | |  | | |  | | | |  | |  | | | | |  |  |  |

**Table S2:** Comparative analysis of nucleotide and amino acids identities of full-genome sequenced CIAV isolates compared to other selected referential and Egyptian strains. Nucleotide and amino acid identities of full genome sequenced CIAV isolates compared to other selected strains, including vaccinal strains. The table displays a comparative alignment in which the nucleotide and amino acid similarity percentages of five Egyptian isolates (genotype II) of 86% -100%. The blue color indicates the commercial CIAV vaccines. The gray color indicates the five Egyptian isolates.

|  | Total body weight(g) | | Thymus weight(g) | | Spleen weight(g) | | Bursa weight(g) | |
| --- | --- | --- | --- | --- | --- | --- | --- | --- |
| Days post infection | **Control group** | **Infected group** | **Control group** | **Infected group** | **Control group** | **Infected group** | **Control group** | **Infected group** |
| 3^rd^ | 71.67±.88 | 56±.88 | 0.32±0.01 | .13±0.00**^***^** | 0.23±0.015 | 0.075±0.001**^***^** | 0.086±0.003 | 0.035±0.001**^**^** |
| 7 ^th^ | 159±.58 | 114.7±.88 | 0.61±0.01 | .32±0.01 **^****^** | 0.44±0.021 | 0.250±0.006**^****^** | 0.33±0.015 | 0.13±0.012**^****^** |
| 14 ^th^ | 471.7±1.67 | 383±2.08**^**^** | 1.14±0.01 | .56±0.01 **^****^** | 0.74±0.010 | 0.570±0.006**^****^** | .39±0.006 | 0.33±0.009**^**^** |
| 21 ^th^ | 923±3.33 | 679.3±2.33**^****^** | 1.65±0.01 | .71±0.01**^****^** | 1.25±0.009 | 0.850±0.0012**^****^** | 1.54±0.021 | 0.52±0.015**^****^** |
| 28 ^th^ | 1555±2.89 | 1106±2.08**^****^** | 2.34±0.01 | 1.15±0.03**^****^** | 1.57±0.012 | 1.053±0.009**^****^** | 1.17±0.012 | 0.47±0.015**^****^** |
| 35 ^th^ | 2552±28.87 | 1750±28.87**^****^** | 2.30±0.06 | 1.42±0.02 **^****^** | 2.32±0.044 | 1.580±0.015**^****^** | 0.84±0.023 | 0.34±0.023**^****^** |
| P Value | <0.0001 | | <0.0001 | | <0.0001 | | <0.0001 | |

**Note:**  Data are presented as mean ± SEM (n=3 per group/time point). Means within the same row (under the same parameter) followed by different lowercase superscripts (a, b) indicate a statistically significant difference (P < 0.05) between the Control and Infected groups

**Table S3.** Comparative analysis of total body weight (g) and absolute lymphoid organ weights (g) in SPF chickens experimentally infected with

the Assiut CIAV Genotype II isolate versus a non-infected control group.

| Blood parameter | Normal range | Control group | Infected group | P value |
| --- | --- | --- | --- | --- |
| Hematocrit (PCV%) | >25% | 32.33±.88 | 18±.58 **^***^** | <0.001 |
| RBCs (10^6/uL) | 2.5-3.5 | 3.5±.29 | 1. 67±.09 **^***^** | <0.001 |
| Hb (g/dL) | 11-13 | 10.67±.88 | 7±.58**^**^** | <0.01 |
| WBC (10^3/uL) | 15-25 | 22.67±1.20 | 12.67±1.76 **^**^** | <0.01 |
| Lymphocytes (%) | 60-70 | 60.00±2.89 | 49.67±.88 **^*^** | <0.05 |
| Heterophils | 20-30 | 30±2.89 | 45.67±1.73 **^**^** | <0.01 |

**Table S4.** Comparative hematological profile at 14 days post-infection (dpi) in SPF chickens experimentally infected with the Assiut CIAV Genotype

Values are expressed as mean ± SEM (n=3 per group). Data were analyzed using an unpaired t-test. Different lowercase superscripts (a, b) within the same row indicate a statistically significant difference (P < 0.05) between the Control and Infected groups.

**Table S5.** Kinetics of humoral immune response (ELISA S/P ratio) in SPF chickens experimentally infected with the Assiut CIAV Genotype II isolate over a 5-week observation period.

| Weeks post infection | Control group (S/P) | Infected group (S/P) | P value |
| --- | --- | --- | --- |
| 1^st^ W | 0.15 ±0.006 | 0.19 ±0.003 | 0.985 |
| 2^nd^W | 0.18 ±0.006 | 1.60 ±0.058******** | <0.0001 |
| 3^rd^W | 0.16 ±0.009 | 0.70 ±0.058 **^***^** | <0.0001 |
| 4^th^W | 0.13 ±0.006 | 0.05 ±0.003 **^**^** | <0.01 |
| 5^th^W | 0.12 ±0.012 | 0.00 ±0.00 | 0.999 |

**Note:** Data are presented as mean S/P ratio ± SEM (n=5 per group). S/P ratios > 0.6 were considered positive according to the manufacturer's threshold (BioChek, Netherlands). Means within the same row with different superscript letters (a, b) are significantly different (P < 0.05) based on Two-way ANOVA followed by Sidak’s multiple comparison test. The rapid decline in S/P ratios in the infected group at 4 and 5 weeks post-infection (wpi) reflects severe immunodepletion, leading to antibody levels falling below the detection limit.

**TableS6.** Dynamics of CIAV viral load (log_10_ DNA copies/mg) in different immune organs of SPF chickens’ post-infection.

|  | Bone marrow | | Thymus | | Spleen | | Liver | |
| --- | --- | --- | --- | --- | --- | --- | --- | --- |
| Days post challenge | **Control group** | **Infected group** | **Control group** | **Infected group** | **Control group** | **Infected group** | **Control group** | **Infected group** |
| 3^rd^ | 0,00±0.0 | 8.67±0.88 **^****^** | 0,00±0.0 | 7.5±0.29 **^****^** | 0,00±0.0 | 6.76±0.44 **^****^** | 0,00±0.0 | 2±0.27 **^****^** |
| 7 ^th^ | 0,00±0.0 | 8.33±0.44 **^****^** | 0,00±0.0 | 8.5±0.29 **^****^** | 0,00±0.0 | 8.03±0.26 **^****^** | 0,00±0.0 | 2.1 ±0.27 **^***^** |
| 14 ^th^ | 0,00±0.0 | 7.5±0.29 **^****^** | 0,00±0.0 | 10±0.29 **^****^** | 0,00±0.0 | 8.8±0. 17**^****^** | 0,00±0.0 | 4±0.27 **^****^** |
| 21 ^th^ | 0,00±0.0 | 4.17±0.44 **^***^** | 0,00±0.0 | 7.8±0.44 **^****^** | 0,00±0.0 | 6±0.58 **^****^** | 0,00±0.0 | 1.17±0.27 **^***^** |
| P Value | <0.0001 | | <0.0001 | | <0.0001 | | <0.0001 | |

**Note:** Viral loads are expressed as mean log_10_ DNA copies per µL ± SEM (n=3 per group/time point). Means within the same row (for each organ) with different lowercase superscripts (a, b) are significantly different (P < 0.05) based on Two-way ANOVA followed by Sidak’s post-hoc test. All control samples remained negative for CIAV DNA throughout the study.


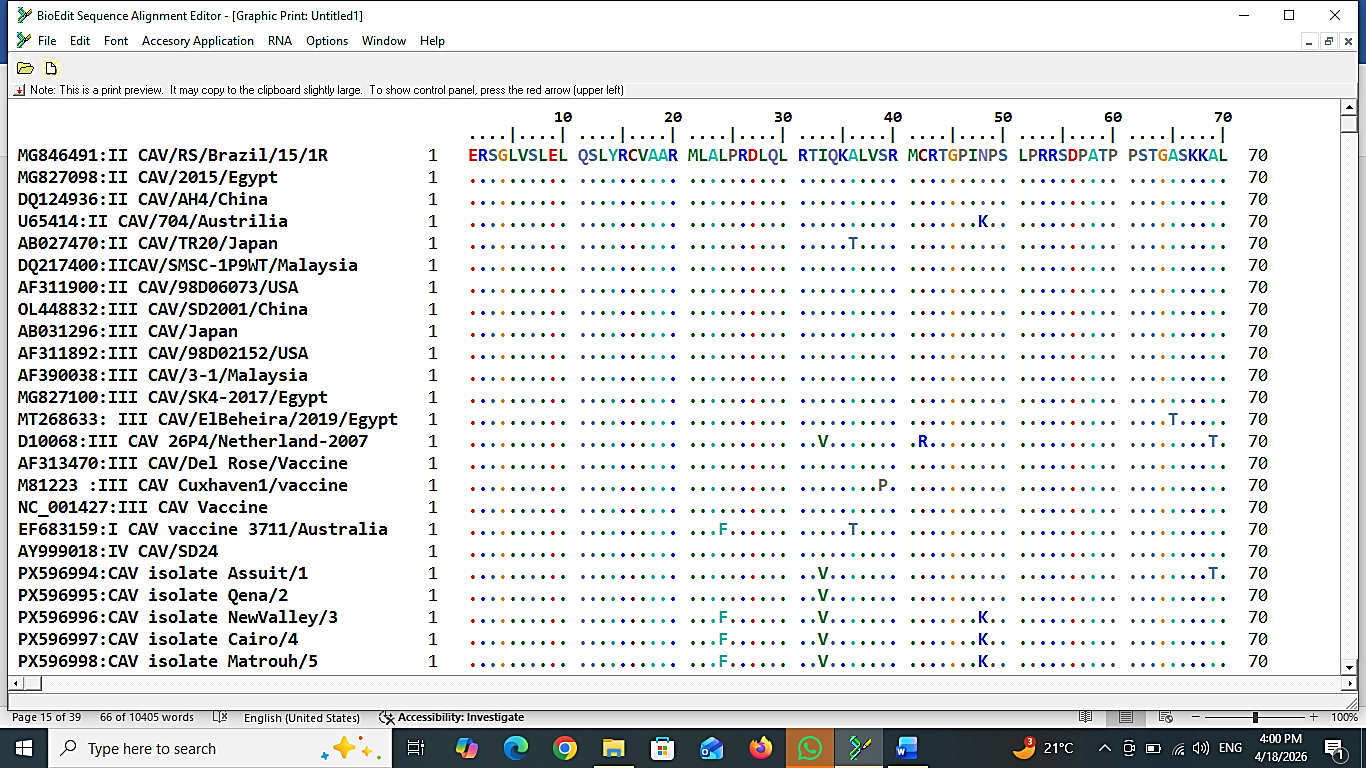

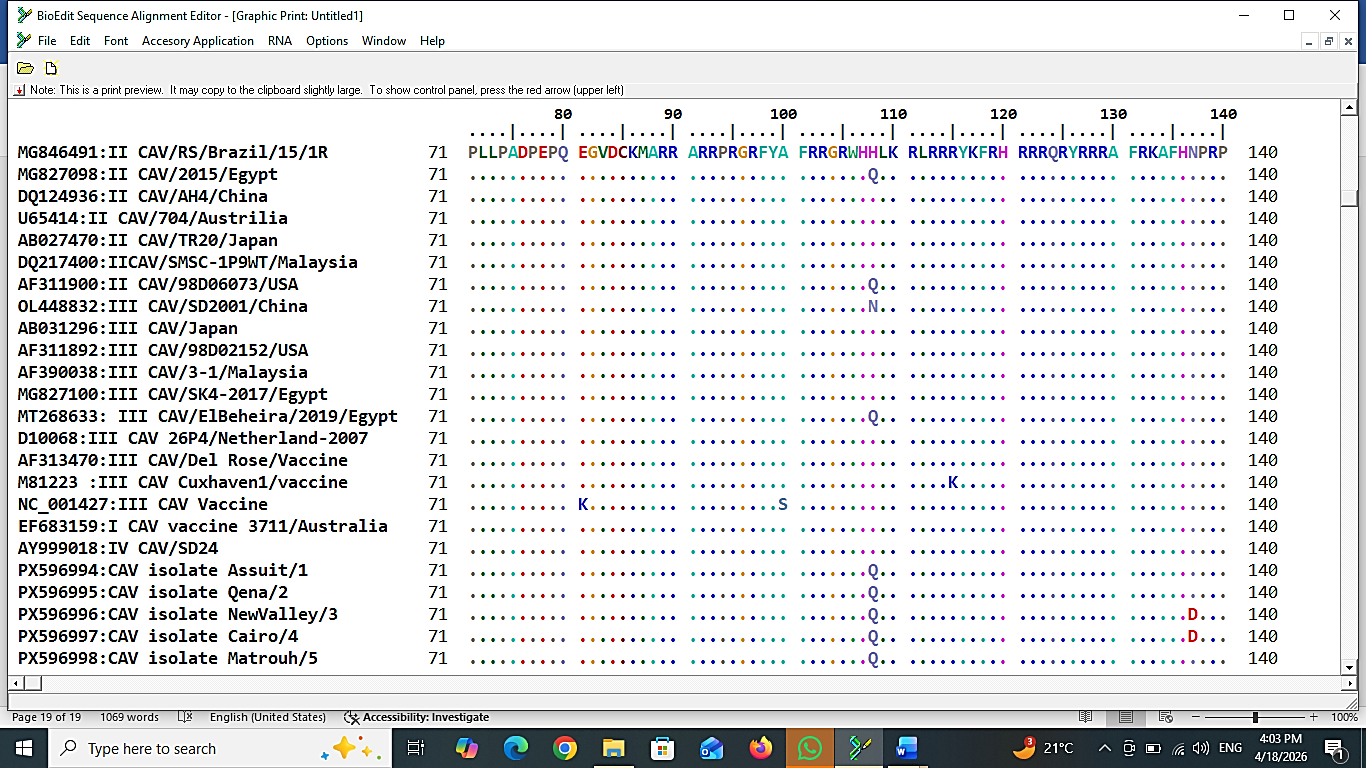

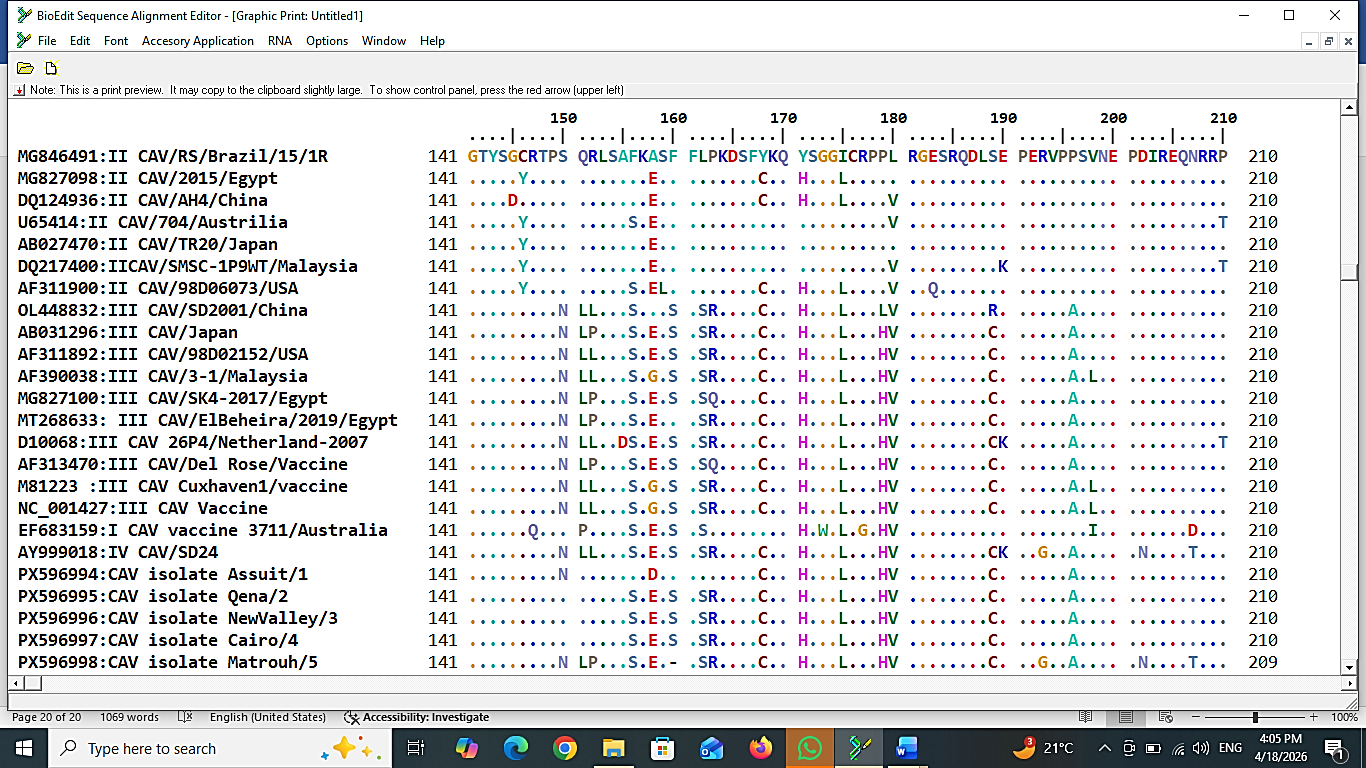


**Figure S1 (A):** Several amino acid sequence alignments of full genomes of CIAV isolates and other reference strains, including Egyptian isolates. The letters denote amino acid substitutions, while dots (.) represent identical amino acids, and dashes (−) indicate alignment.


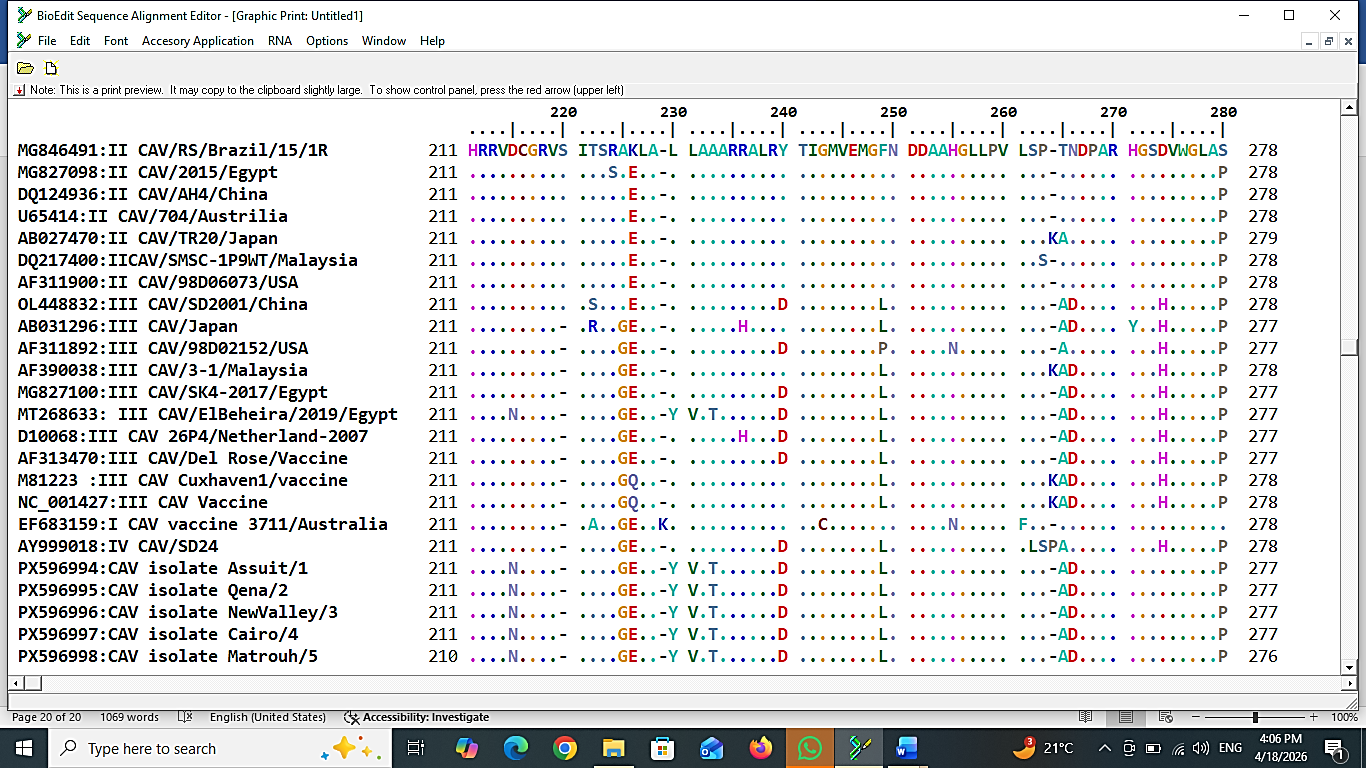

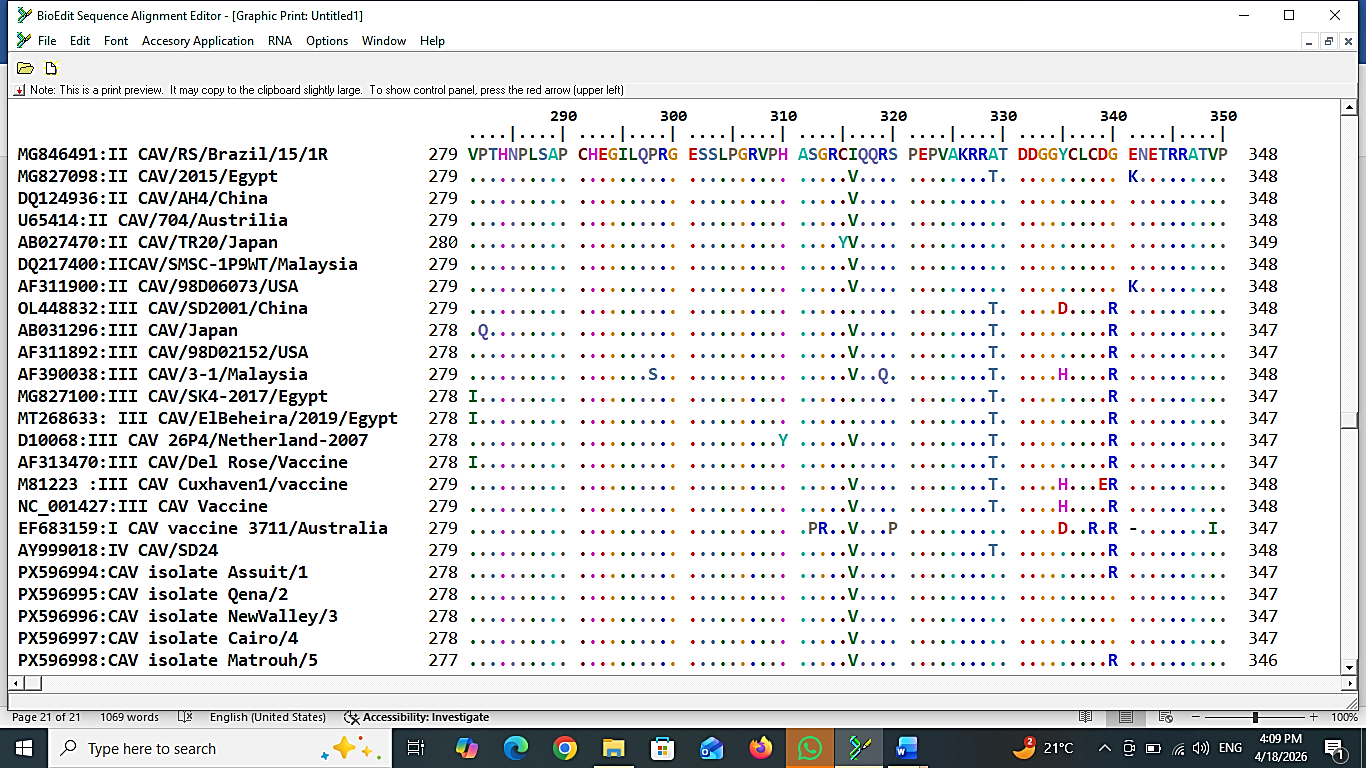

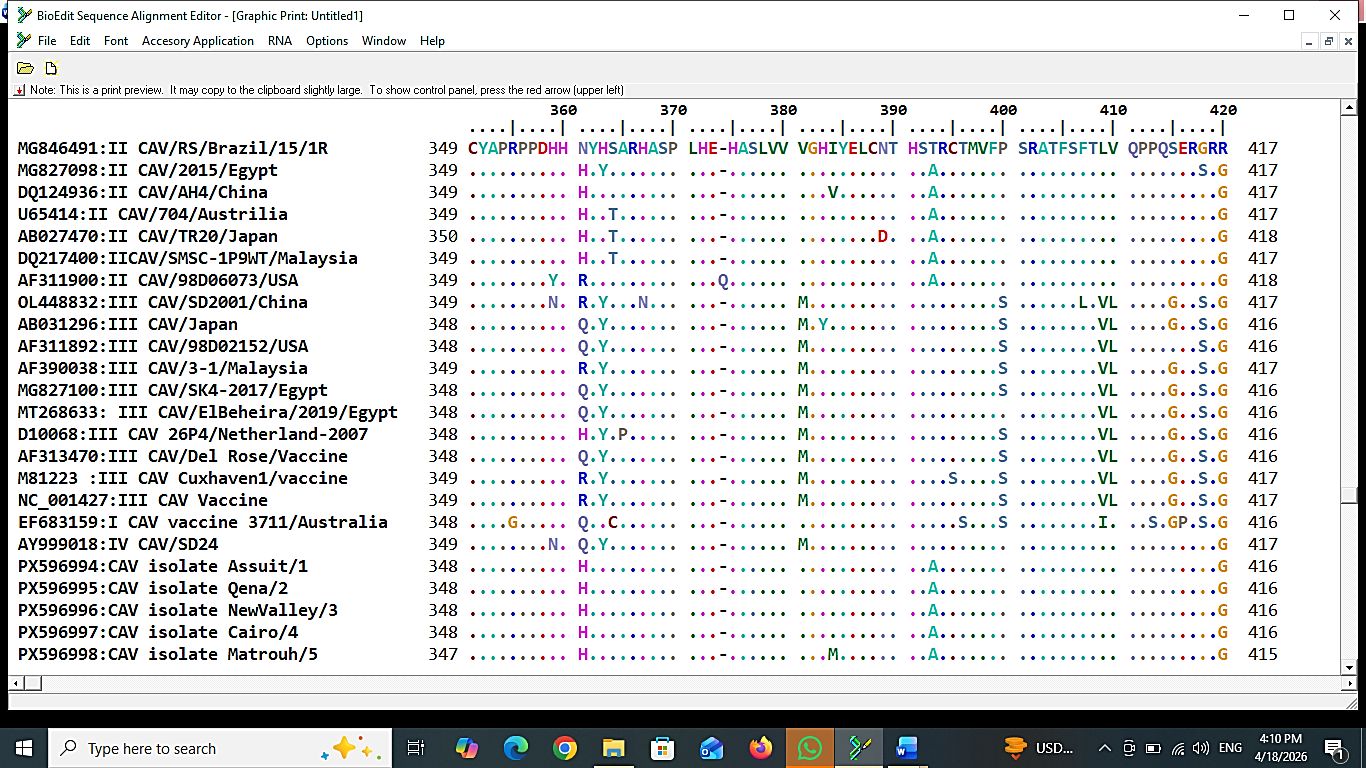


**Figure S1 (B):** Several amino acid sequence alignments of full genomes of CIAV isolates and other reference strains, including Egyptian isolates. The letters denote amino acid substitutions, while dots (.) represent identical amino acids, and dashes (−) indicate alignment.


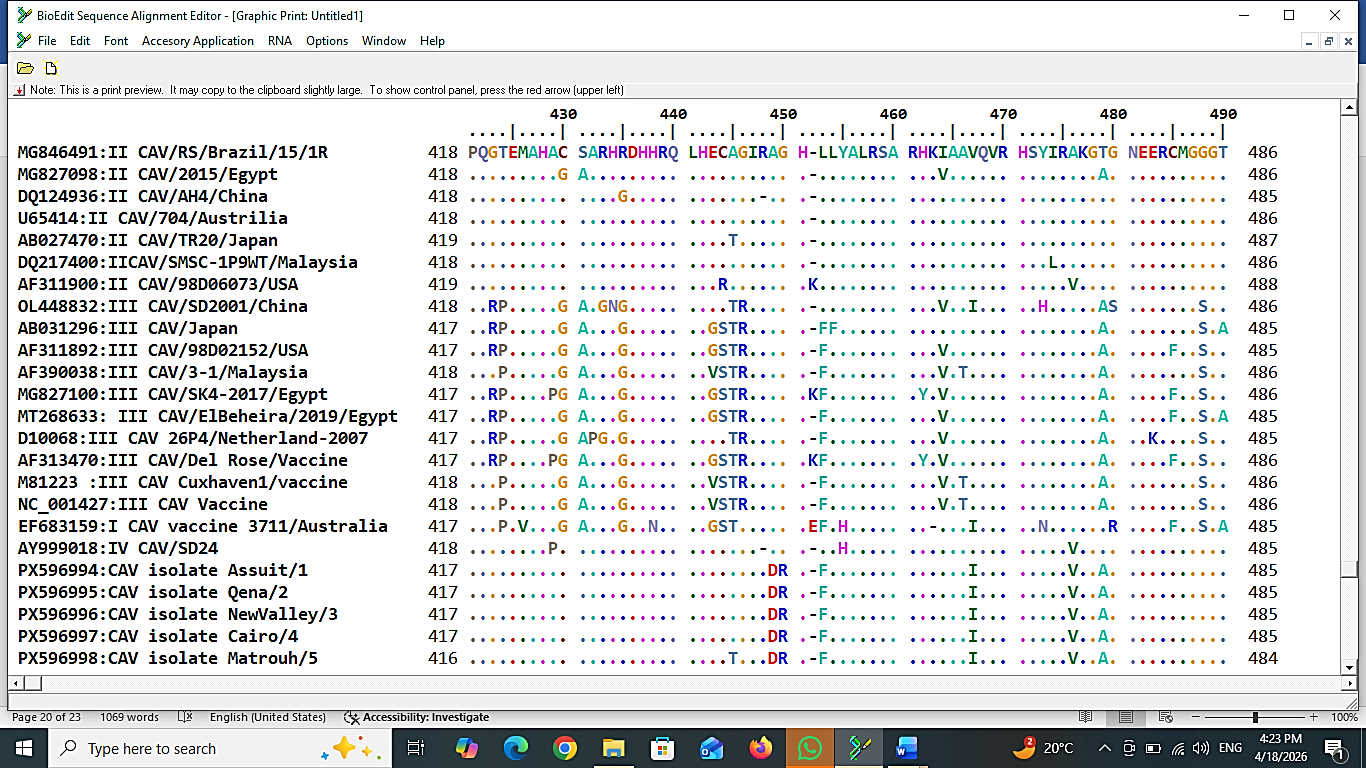

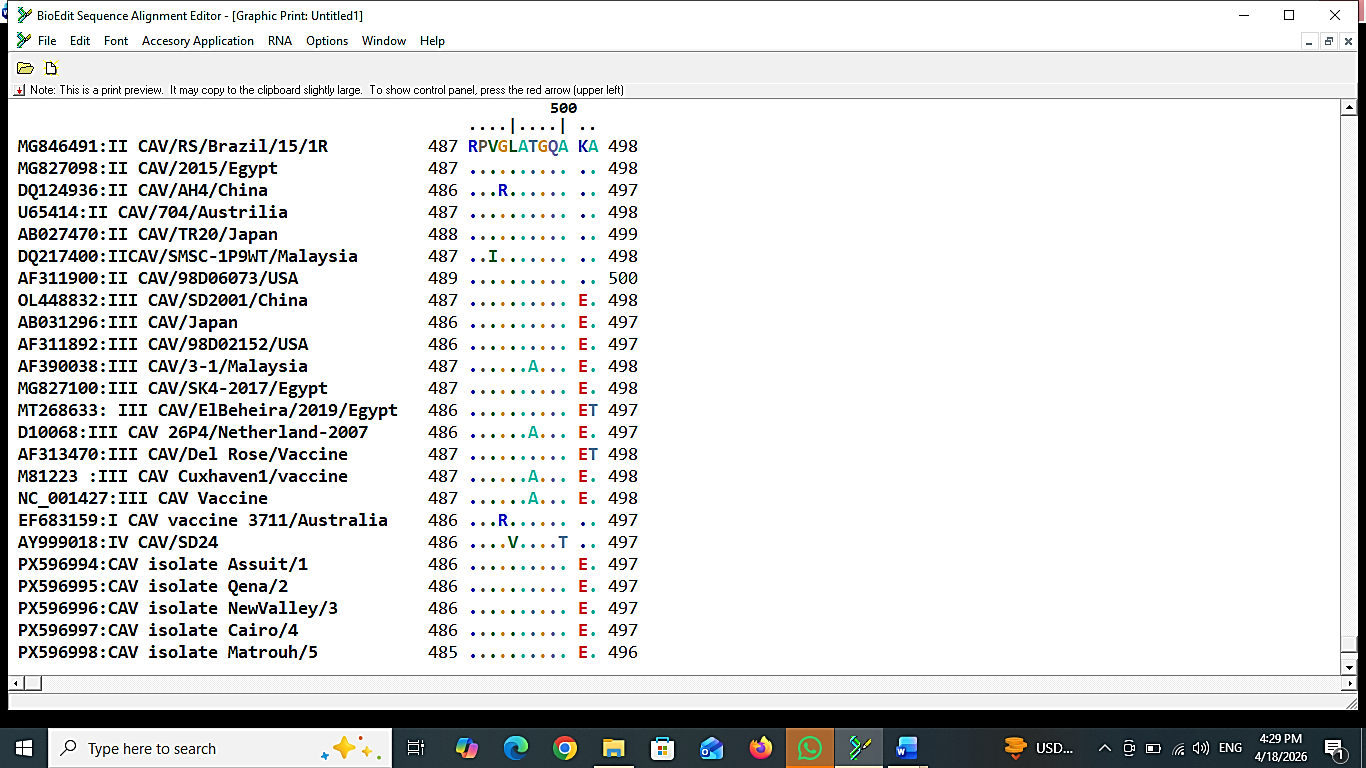


**Figure S1 (C):** Several amino acid sequence alignments of full genomes of CIAV isolates and other reference strains, including Egyptian isolates. The letters denote amino acid substitutions, while dots (.) represent identical amino acids, and dashes (−) indicate alignment.


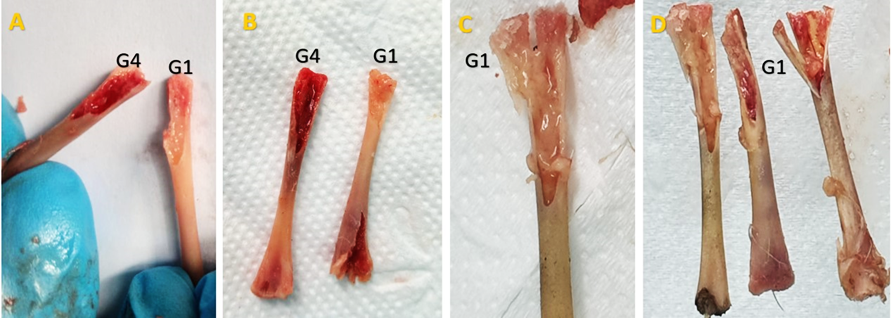

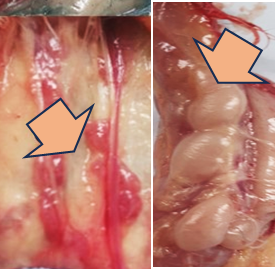

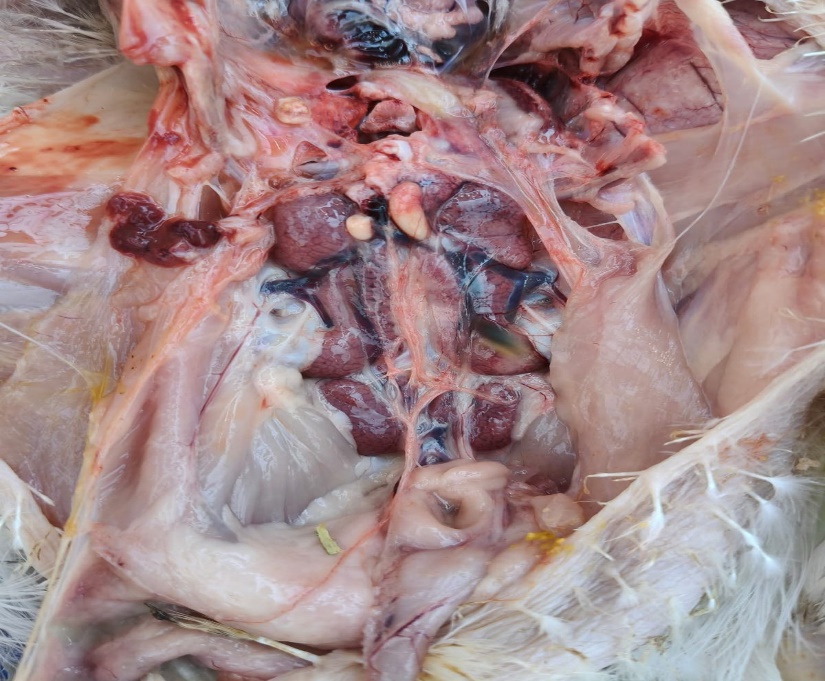

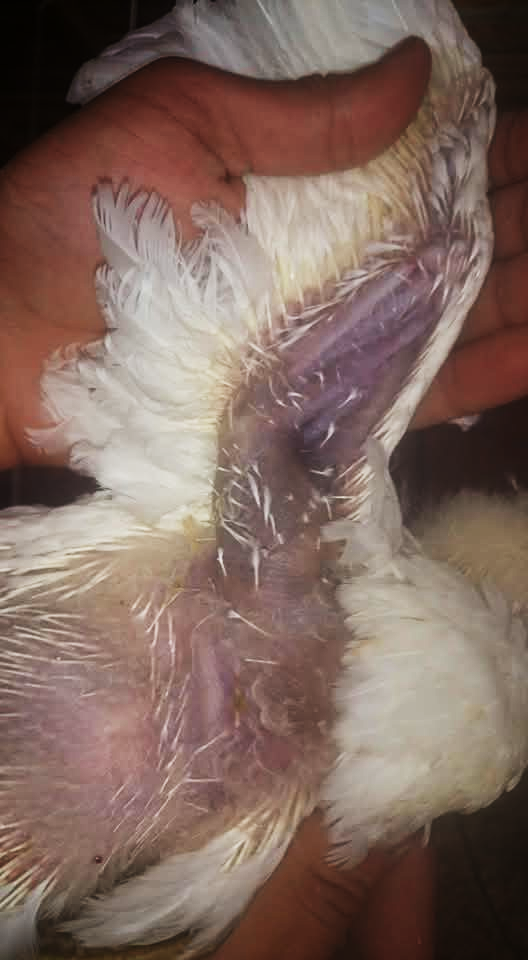

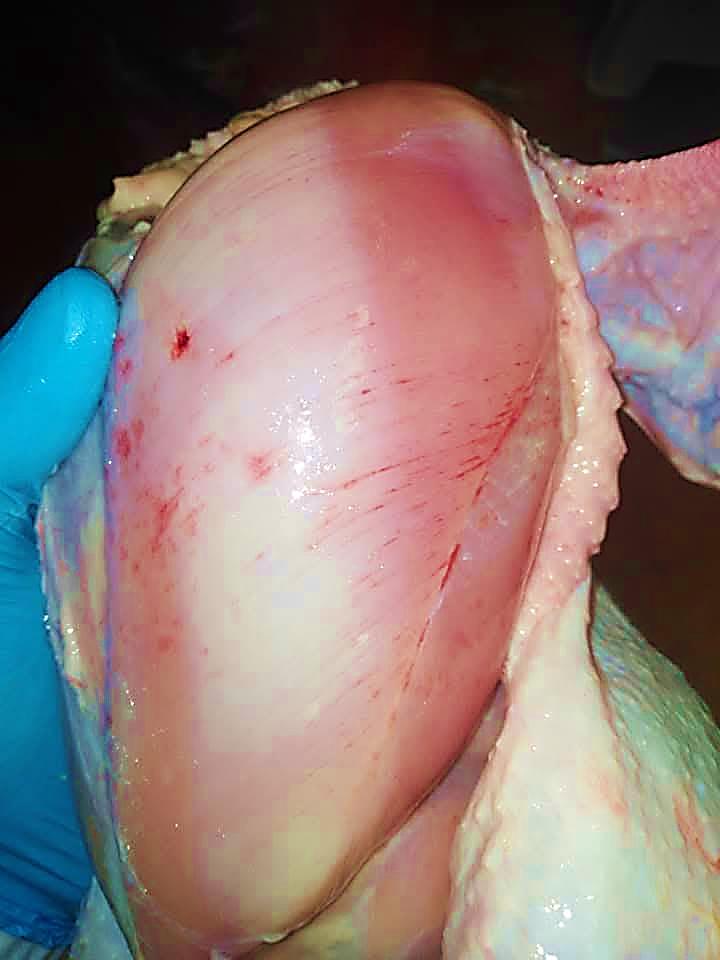

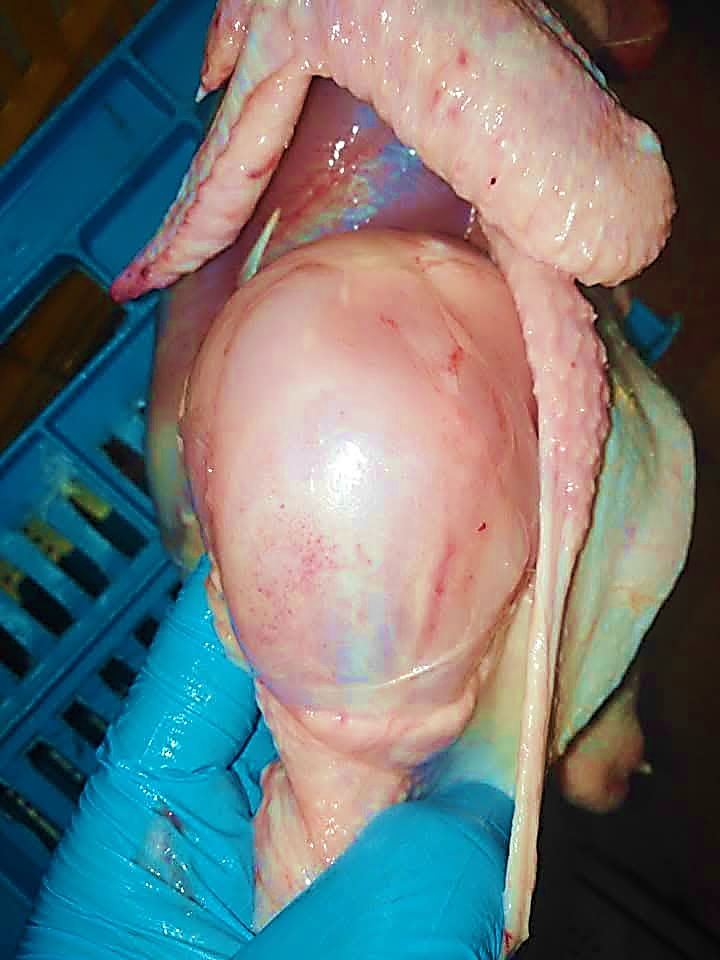


**Figure S2:** Macroscopic post-mortem lesions induced by experimental infection with the Upper Egyptian CIAV Genotype II isolate (Assiut strain). **(A)** Blue-wing disease, showing severe gangrenous dermatitis and extensive cyanosis and hemorrhages on the subcutaneous tissue and skeletal muscles of the wing tip. **(B)** Breast and thigh muscles showing extensive petechial and ecchymotic hemorrhages. **(C)** Severe glandular mucosal hemorrhages on the proventriculus at the junction with the gizzard. **(D)** Cross-sections of femurs. Left: Pale, fatty bone marrow indicative of severe aplastic anemia; Right: Normal deep red hematopoietic bone marrow from a control chick. **(E)** Severe atrophy of the thymus lobes (white arrows indicate involuted chains, peach arrow highlights the extremely small size of the lobes) and atrophied bursa of Fabricius. **(F)** Enlarged and distinctly pale kidneys with severe urate deposition in the ureters (nephrosis), a direct consequence of systemic immunopathology.


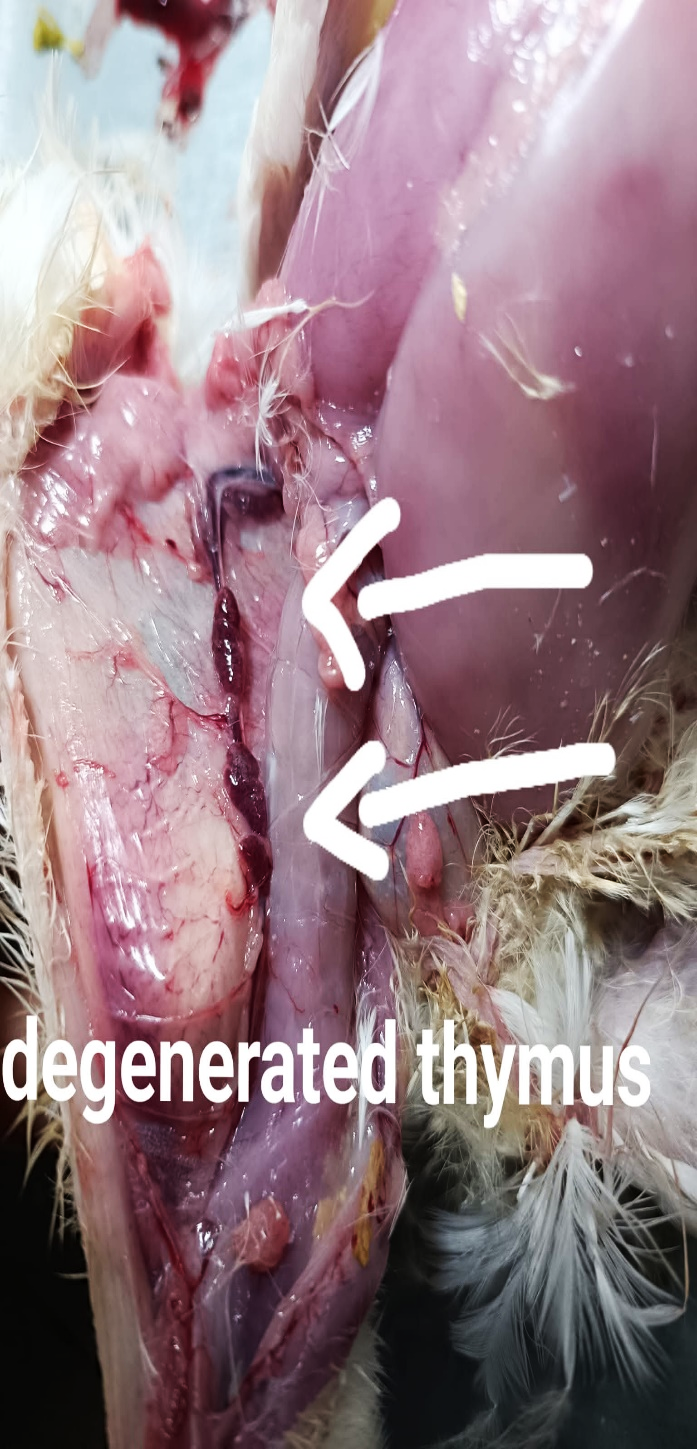


**A**

**B**

**D**

**E**

**F**


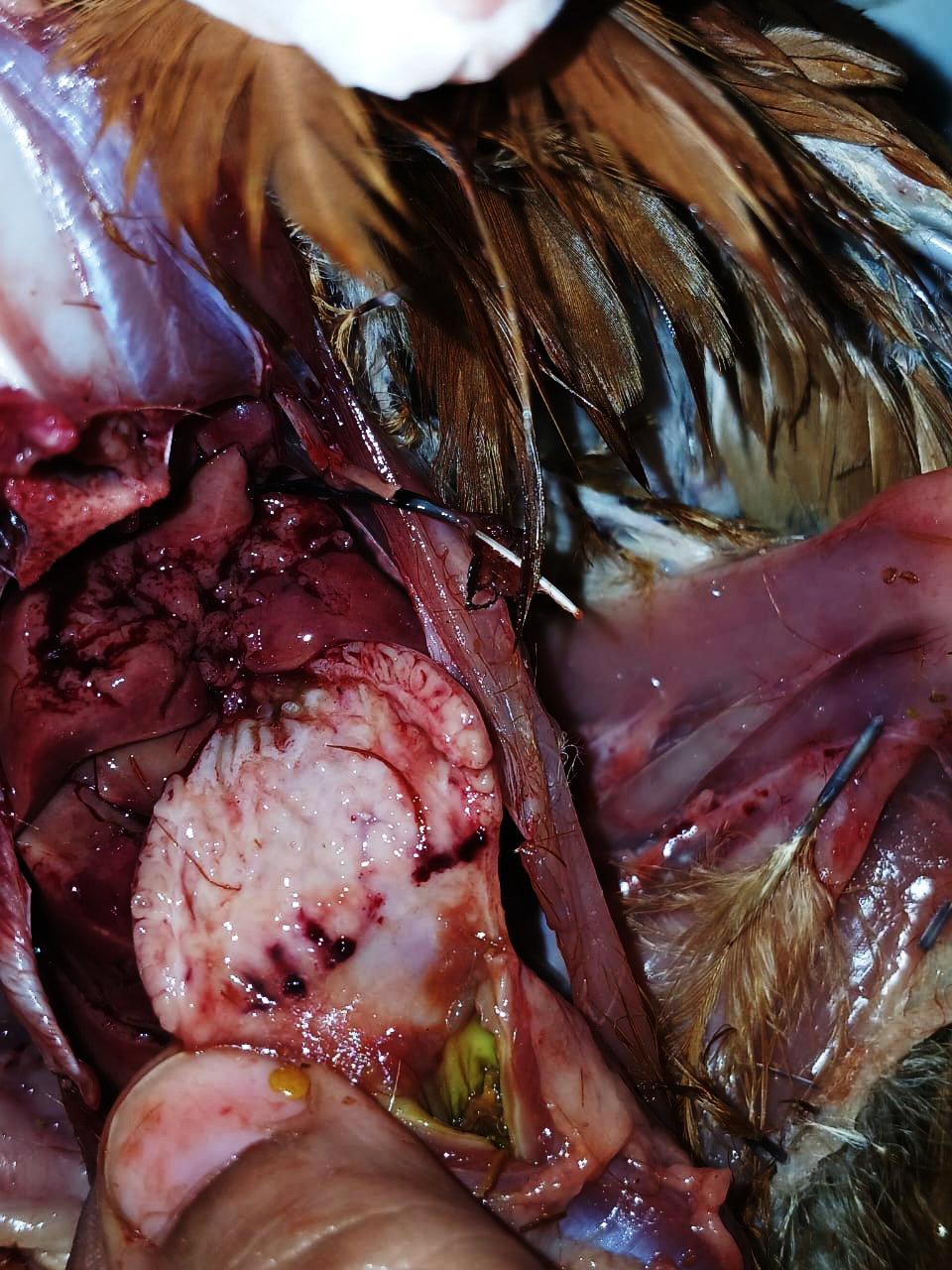


**C**
